# ISGylation is induced in neurons by demyelination driving ISG15-dependent microglial activation

**DOI:** 10.1186/s12974-022-02618-4

**Published:** 2022-10-20

**Authors:** Benjamin D. S. Clarkson, Ethan Grund, Kenneth David, Renee K. Johnson, Charles L. Howe

**Affiliations:** 1 Department of Neurology, Mayo Clinic, Rochester, MN 55905 USA; 2 Department of Laboratory Medicine and Pathology, Mayo Clinic, Guggenheim 1521C, 200 First Street SW, Rochester, MN 55905 USA; 3 Concordia College, Moorhead, MN USA; 4 Division of Experimental Neurology, Mayo Clinic, Rochester, MN 55905 USA; 5 Center for Multiple Sclerosis and Autoimmune Neurology, Mayo Clinic, Rochester, MN 55905 USA; 6 Mayo Clinic Graduate School of Biomedical Sciences, Mayo Clinic Alix School of Medicine and Mayo Clinic Medical Scientist Training Program, MN 55905 Rochester, USA

**Keywords:** ISGylation, ISG15, Multiple sclerosis, Extracellular vesicles, Exosomes, miRNA

## Abstract

The causes of grey matter pathology and diffuse neuron injury in MS remain incompletely understood. Axonal stress signals arising from white matter lesions has been suggested to play a role in initiating this diffuse grey matter pathology. Therefore, to identify the most upstream transcriptional responses in neurons arising from demyelinated axons, we analyzed the transcriptome of actively translating neuronal transcripts in mouse models of demyelinating disease. Among the most upregulated genes, we identified transcripts associated with the ISGylation pathway. ISGylation refers to the covalent attachment of the ubiquitin-like molecule interferon stimulated gene (ISG) 15 to lysine residues on substrates targeted by E1 ISG15-activating enzyme, E2 ISG15-conjugating enzymes and E3 ISG15-protein ligases. We further confirmed that ISG15 expression is increased in MS cortical and deep gray matter. Upon investigating the functional impact of neuronal ISG15 upregulation, we noted that ISG15 expression was associated changes in neuronal extracellular vesicle protein and miRNA cargo. Specifically, extracellular vesicle-associated miRNAs were skewed toward increased frequency of proinflammatory and neurotoxic miRNAs and decreased frequency of anti-inflammatory and neuroprotective miRNAs. Furthermore, we found that ISG15 directly activated microglia in a CD11b-dependent manner and that microglial activation was potentiated by treatment with EVs from neurons expressing ISG15. Further study of the role of ISG15 and ISGylation in neurons in MS and neurodegenerative diseases is warranted.

**Supplementary Information:**

The online version contains supplementary material available at 10.1186/s12974-022-02618-4.

## Background

Grey matter (GM) pathology in multiple sclerosis (MS) includes gliopathy, demyelination, neuron loss, atrophy, limited recruitment of peripheral immune cells, prominent immune cell recruitment in adjacent meningeal structures (for subpial lesions that extend into GM), and selective synapse loss—so-called synaptopathy [1–14]. Indeed, many of these features are present outside of GM lesions in ‘normal appearing’ GM—suggesting that these phenomena are independent of local demyelination. Recent studies have also demonstrated that GM pathology in both cortical GM and deep GM structures is present in the earliest clinical phases of MS [15–21] and may be present in clinically and radio-graphically isolated syndrome patients that later develop clinically definite MS [22–31]. Together with evidence that measures of GM atrophy and neuron loss tend to be among the stronger correlates of irreversible disease progression in MS [32–37], these data suggest that GM pathology drives progression in both early and late MS and underlies a large part of the clinical burden of MS.

Evidence suggests that anterograde and retrograde axonal stress signals arising from white matter lesions could promote diffuse GM pathology, atrophy, and new lesion formation. To identify the most upstream transcriptional responses arising in neurons from these anterograde and retrograde signals, we used synapsin-CRE mice crossed with RPL22-floxed ‘ribotag’ mice to analyze ribosome-associated transcripts from cortical neurons isolated from mice with experimental autoimmune encephalomyelitis (EAE) or cuprizone-induced demyelination (Clarkson et al. 2022a *under review*). In addition to increased expression of MHC I genes and related antigen processing and presentation genes, we found elevated levels of transcripts associated with the ISGylation pathway. This suggested that ISGylation induction might be a shared stress response in neurons following both chemical and inflammatory demyelination. ISGylation refers to the covalent attachment of the ubiquitin-like molecule interferon stimulated gene (ISG) 15 to lysine residues on substrates targeted by E1 ISG15-activating enzyme, E2 ISG15-conjugating enzymes and E3 ISG15-protein ligases often co-translationally. Unlike ubiquitin, ISG15 is not constitutively expressed in most cells and rather than promoting proteasomal degradation of targeted proteins, attachment of ISG15 to target proteins proteasome dependent degradation. In fact, ISG15 is unknown to interrupt the formation of poly-ubiquitin chains by directly conjugating to lysine residues on ubiquitin and instead regulates ubiquitin-dependent targeting of proteins for proteasomal degradation. ISGylation been implicated in regulation of mitophagy, autophagosome formation, autophagic flux [38–42]. In immune cells, ISG15 has been chiefly described for its capacity to neutralize or tag viral proteins, prevent ubiquitin-mediated targeting of viral sensing proteins to the proteasome[43, 44], block exosome secretion [45], promote lymphocyte activation and contribute to viral control [43, 46–52]. However, its role in neurons is not well established. Therefore, we sought to identify possible upstream signals that drive ISG15 expression in neurons and to determine the functional consequences for cortical neurons and glial cells when neurons exhibit elevated levels of ISG15 expression and ISGylation.

## Methods

### Mice

C57BL/6 wild-type (WT, stock# 000664), B6(Cg)-Ifnar1tm1.2Ees/J (IFNAR KO, stock#028,288), B6.Cg-Tg(Syn1-cre)671Jxm/J (Syn.Cre, stock# 003966), and B6J.129(Cg)-Rpl22tm1.1Psam/SjJ (Rpl22, stock #029,977) were obtained from the Jackson Laboratory (Bar Harbor, ME). Syn.Cre + / + mice were crossed with RPL22 + / + mice to generate Syn.CrexRPL22 F1 mice used for ribotag experiments. All F1 offspring used in experiments were screened for CRE by PCR on gDNA isolated from tail clippings using the following primers: Syn.Cre-forward IMR1084 GCGG TCTG GCAG TAAA AACT ATC, Syn.Cre-reverse IMR1085 GTGA AACA GCAT TGCT GTCA CTT, positive internal control forward IMR7338 CTAG GCCA CAGA ATTG AAAG ATCT, positive internal control reverse IMR7339GTAG GTGG AAAT TCTA GCAT CATC C. All animal experiments were approved by the Mayo Clinic Institutional Animal Care and Use Committee in accordance with National Institutes of Health guidelines.

### *Immunohistochemistry and *in situ* hybridization*

After deparaffinization and rehydration, sections were treated with 3% hydrogen peroxide in Tris-buffered saline (TBS) for 30 min and then underwent heat-induced antigen retrieval in Tris–EDTA (10 mM/1 mM; pH 9.0) or citrate (10 mM pH 6.0) for 30 min at 95C. Sections were blocked for 30 min with 3% bovine serum albumin and 2.5% secondary serum in TBS and then stained with primary antibodies, 5 µg/mL rabbit anti-ISG15 (1H9L21), 1:400 mouse anti-HLA-DR (LN3) or 1:500 mouse-anti-PLP (plpc1) overnight at 4 °C. Protein adsorption was used as a negative control. After several washes, secondary antibodies (biotin-SP donkey anti-rabbit or biotin-SP donkey anti-mouse) were applied to sections and incubated for 2 h at room temperature. Staining was developed using the VECTASTAIN® Elite® ABC HRP Kit (Vector Laboratories) with 0.5 mg/mL diaminobenzidine (Sigma) and 0.01% H2O2. Slides were lightly counterstained with hematoxylin, rinsed with running tap water, and mounted with toluene. Automated in situ hybridization was performed by the Mayo Clinic Pathology Research Core following optimization and validation using the following RNAscope 2.5 LS Probes (ACD Bioteche) with RNAscope 2.5 Reagent Kit Brown (#322100) in accordance with the manufacturer’s instructions: Hs-USP18-O1 (#516,328), Hs-HERC5 (#495,548). Hs-PPIB (#313908) and dap-B (#312038) probes were used as positive and negative probes on all tissues to confirm RNA integrity and sample quality. Images were acquired on a CKX41 microscope equipped with a CP73 camera (Olympus) using Cell View image acquisition software. Digital images were processed and analyzed using ImageJ software. Micrographs with excessive background or tissue fRolding were excluded from subsequent analyses. ISG155 staining intensity was qualitatively scored on coded images using the following scale: 0 = no staining, 1 = intermittent light staining (1–3 cells per field), 2 = pervasive light staining (> 3 cells per field), 3 = intermittent dark staining, 4 intermittent dark staining with pervasive light staining, 5 = pervasive dark staining. Average values (representative of 2–3 micrographs) are reported for each patient. For semiquantitative analysis of in situ hybridization digital images were processed and analyzed using Fiji ImageJ software as previously described (Crowe and Yue 2019). Briefly, following image deconvolution, and DAB channel thresholding, mean staining intensity was measured. Average values (representative of 5–7 micrographs) are reported for each patient. All contrast manipulations were applied equally to each image.

### Cortical neuron cultures and microfluidic devices

Tissue-culture treated vessels were coated with 0.5 mg/mL poly-ornithine in 100 mM borate buffer. Cortical neurons were prepared from C57BL6 mouse embryonic day 15 (E15) pups as previously described [53, 54]. Briefly, the upper halves of the cerebral cortices were dissected, and meninges were removed and washed in Hank’s Balanced Salt Solution (HBSS). The tissue was washed in neuron plating media, which contained high-glucose Dulbecco’s modified Eagle medium (DMEM) with glutamine, supplemented with 10% bovine calf serum and 10% F12. Cortices were digested in 2 mg/mL papain in HBSS at 37 °C for 15 min, triturated with 5 ml serological pipet, p1000 pipet tip, glass Pasteur pipet and finally fire polished glass Pasteur pipet until cortices were dissociated into a single-cell suspension. Cells were centrifuged at 400*g* for 4 min and seeded at 4.5 × 10^5^ cells per cm2 (10^6^ cells per mL) on poly-ornithine coated plates. In some experiments, cells were infected at plating with 2000 MOI (2 × 10^9^ gc/mL) of various adeno-associated viruses (AAVs). Three hours after seeding, the cortical neurons were fed with 2 volumes neuron feed media containing Neurobasal media supplemented with 2% B27, 1% Glutamax, and 100 U/mL penicillin and streptomycin. During the first 48 h cells were given 1 ng/mL brain-derived neurotrophic factor (BDNF) and 10 ng/ml insulin-like growth factor 1 (IGF1). Subsequently, neurons were maintained in neuron feed media by changing half media volume every 2–3 days. In some experiments neurons were cultured in polydimethylsiloxane (PDMS) microfluidic axon isolation chambers as previously described [53]. PDMS microfluidic chambers were fabricated by pouring PDMS (Sylgard184 Elastomer, Dow Inc.) plus 10% v/v crosslinking reagent onto silicon wafer molds generated by soft lithography (APP Systems). Dimension of these chambers are described elsewhere [55]. Neuronal progenitor cells (2 × 10^5^) were plated in soma chambers in plate media and fed 3 h later with neuron feed media plus BDNF and IGF1 as above. Neuron feed media without BDNF and IGF was replaced every 2–3 days on the soma chamber and media containing BDNF and IGF1 was added every 2–3 days to the distal chamber to support trophic factor-driven directional outgrowth of axons into the distal chamber.

### Western blot analysis

For Western blot analysis mouse cortical neuron cultures were infected at plating with AAV vectors. Protein lysates were prepared from these cultures at DIV 12–14 using lysis buffer containing Nonidet P40 (1% v/v), deoxycholic acid sodium salt (0.5% w/v), and glycerol (10% v/v) plus the protease inhibitors NaF (1 mM), NaVO_3_ (1 mM), Aprotinin (10 µg/mL), Leupeptin (1 µg/mL), and phenylmethylsulfonyl fluoride (1 mM). Lysates were denatured for 5 min at 95 C in Laemmli buffer (1:1) and 30 uL loaded into each well of a 4–20% criterion gel. After electrophoresis, proteins were transferred to polyvinylidene difluoride membrane, blocked for 1 h with 5% bovine serum albumin in TBS, and probed with primary mouse antibodies (clone, 1:2000) in block buffer overnight at room temperature. Afterward blots were washed and probed with secondary antibody (horse radish peroxidase conjugated donkey anti-mouse; 1:5000 Jackson Immunolab) in block buffer for 60 min at room temperature. After extensive washing blots were developed with SuperSignal West Pico Plus Chemiluminescent Substrate and acquired on a ChemiDoc (BioRad) by luminescence imaging or CL-XPosure™ Film on X-OMAT (Kodak). Blots were stripped with pH 1.85 TBS for 10–15 min for re-probing with anti-beta actin primary (1:5000) for loading control following the protocol above.

### Cuprizone, EAE, and pertussis toxin injections

Experimental autoimmune encephalomyelitis (EAE) was induced as previously described [56–58]. Briefly, emulsion of equal volumes of CFA and 100 µg myelin oligodendrocyte glycoprotein peptide (MOG_35–55_, MEVGWYRSPFSRVVHLYRNGK) supplemented with *M. tuberculosis* H37Ra (5 mg/mL, Difco, Detroit, MI) were injected subcutaneously in the scapular region of each mouse. MOG–CFA mixture was emulsified by sonication using a sonic dismembrator (Fisherbrand; FB705). Pertussis toxin (200 ng/mouse, i.p.; List Biological Laboratories) was injected on the days 0 and 2 relative to immunization. Clinical scores were monitored daily in a blinded manner and recorded as follows: 0, no clinical disease; 1, flaccid tail; 2, gait disturbance or hind limb weakness; 3, hind limb paralysis and no weight bearing on hind limbs; 4, hind limb paralysis with forelimb paresis and reduced ability to move around the cage; and 5, moribund or dead. Only animals exhibiting clinical disease were examined by transcriptional analysis. For experiments modeling blood brain barrier disruption, 200 ng pertussis toxin was injected i.p. in 500 µL PBS as above 96 h and 48 h prior to adoptive transfer of CD8 + T cells. Cuprizone (Bis(cyclohexanone)oxaldihydrazone; Sigma) diet containing 0.3% w/w cuprizone was prepared by Test Diet in 5LG6 base diet and utilized within 6 months of manufacture. For experiments, mice were allowed access to experimental or control (5LG6) diet ad libitum and monitored weekly for 6 weeks prior to further manipulation.

### Ribotag translational profiling

For translational profiling experimental SynCre.Rpl22 mice were perfused transcardially with cold PBS containing 20 ug/mL cycloheximide and 0.1 mg/mL heparin and subsequently process as previously described to isolate neuronal ribosome-bound mRNA [59]. Briefly, mouse cortex was grossly dissected and homogenized with sterile RNase-Zap treated Dounce homogenizers in 10% w/v homogenization buffer containing 45 mM Tris, 100 mM KCl, 12 mM MgCl2, 1% NP-40, 20 ug/mL cycloheximide, 1 mg/mL heparin, 0.5% RNasin, 1% protease inhibitor cocktail (Sigma) and 1 mM DTT. Homogenate was centrifuged for 20 min at 5000 g and supernatant aliquoted for storage at – 80 °C. Later 7.5 ug/mL HA1.1 mAb was added to homogenate and incubated with gentle agitation for 4 h at 4C. Subsequently, 0.25 volumes Dyna Protein G beads were resuspended in homogenization buffer and added to homogenate for overnight incubation. The next day samples were placed on an EasySep magnet (StemCell Technologies) for 3–5 min and negative fraction was removed. Beads were washed 3 times with high salt buffer containing 45 mM Tris, 300 mM KCl, 12 mM MgCl2, 1% NP-40, 20 ug/mL cycloheximide and 1 mM DTT. Lastly, mRNA was eluted off bead-bound ribosomes by resuspending the beads in Buffer RLT Plus (Qiagen) and vortexing vigorously. RNA was then isolated using RNeasy Micro Plus spin column assembly following manufacturer’s instructions (Qiagen).

### RT-PCR and microarray analysis

Cell lysates were dissociated and homogenized using QiaShredder (Qiagen), following the manufacturer’s instructions. RNA was isolated using RNeasy micro plus kit (Qiagen) and genomic DNA was excluded using gDNA eliminator spin column. Relative expression of the indicated genes was quantitated relative to the house keeping gene GAPDH. Gene specific primers (Additional file 1: Table S1) were selected using NCBI Primer Blast and 20 µL reactions using SsoAdvanced Universal SYBR Green Supermix (BioRad) were carried out following the manufacturer’s instructions. Ribotag microarray analysis was performed at the University of Illinois at Chicago Core Genomics Facility. Briefly, RNA was isolated from neuronal ribosomes as described above and quality control was performed using an Agilent TapeStation system. Total RNA was then used to create the biotin-labeled library to be hybridized on GeneChip® Mouse Gene 2.0 ST Arrays (Affymetrix) microarrays containing the entire mouse transcriptome (34 K transcripts) including ~ 26,500 coding and > 3500 non-coding transcripts. Following the manufacturer's protocols a total of 16 microarray chips were used for 6 EAE, 6 Cuprizone and 4 control samples with quality control verification before amplification and before hybridization. The arrays were finally scanned in an Affymetrix GeneTitan System. Intensities of target hybridization to respective probe features were detected by laser scanning of the array. Raw data (CEL files) were imported into Transcriptome Analysis Console (Affymetrix) and normalized by robust multiarray average (RMA). Statistical analysis was performed considering an FDR < 20%.

### Tissue processing for immunofluorescence and confocal imaging

For immunostaining, mice were deeply anesthetized and then perfused transcardially with > 15 mL heparinized saline solution followed by > 30 mL 4% paraformaldehyde (PFA) solution. Brains and cervical lymph nodes were collected and further post fixed in 4% PFA for 24 h. Brains were then washed and transferred to 30% sucrose solution prior to embedding. For thick sections, brains were embedded in 4% low melting point agarose in PBS and 70-micron floating sections were cut on VT1000 P Manual vibratome (Leica). Floating sections were permeablized overnight in 0.1% Triton-X 100 in TBS and then transferred to blocking buffer containing 2.5% normal donkey serum, 5% bovine serum albumin, and 0.1% Triton-X 100 in TBS for 2 h. Floating sections were then transferred to blocking buffer containing primary antibodies (5 ug/mL), washed again and counterstained with DAPI prior to mounting with VECTASHIELD Antifade Mounting Media (Vector Biolabs). For thin sections (8–20 micron), tissues were instead embedded in cryomolds with optimal cutting temperature compound. Sections were cut at -18C to -20C on a cryostat, transferred to subbed slides and immunostained as above. For CD8 staining, sections were not permeablized and Triton-X 100 was excluded from staining and wash buffers.

### Proteomic analysis

For immunoprecipitation experiments, mature cultures of human neuronal cultures were lysed in NDG lysis buffer containing 1% Nonidet P-40 (v/v), 0.5% deoxycholic acid sodium salt (w/v) and 10% glycerol (v/v) in 1X Tris-buffered saline (20 mM Tris, 137 mM NaCl, pH 8.0 in ddH2O), 1 mM PMSF, 10 μg/mL aprotinin, 1 μg/mL leupeptin, 1 mM NaVO3 and 1 mM NaF. Lysates were immunoprecipitated with biotinylated anti-ISG15 (clone F9) using streptavidin magnetic nanoparticles (COMPANY). Proteins were eluted in 5% glacial acetic acid, dried and resuspended in HPLC grade H2O. Eluates were digested with trypsin, desalted, and reconstituted in 0.1% formic acid for injection. Samples were analyzed using nano high-pressure liquid chromatography electrospray tandem mass spectrometry (Mayo Clinic Medical Genome Facility, Proteomics Core). For EV experiments, EVs were purified from cell supernatant by ultracentrifugation, lysed as above and dried EV lysates were used for mass spectrometry. Human protein identification was performed using UniProt Reviewed 2019_04 Human database with reverse decoy sequence. Mouse protein identification was performed using UniProt Reviewed 2019–05 Mouse database with reverse decoy sequence. Protein identification criteria were > 95% probability; 2 peptides minimum; 95% peptide threshold. False positivity rate was 0.4%–0.7%.

### Cytometric bead array analysis

Cytometric bead array analysis of proinflammatory cytokines was performed on a C6-Accuri flow cytometer (BD) using Mouse Inflammation CBA kit (BD) according to the manufacturer’s instructions. Briefly, 50 uL cell supernatants or standards were incubated for 2 h at room temperature with 50 uL containing each of 6 capture beads against IL10, IL12p70, TNFa, CCL2, IFN*γ*, and IL6. PE-detection reagent containing PE-labeled antibodies against the cytokines above was then added to these samples (50 uL) for another 2 h, after which samples were spun down, washed, and acquired. Analysis was performed in FCAP array v3.0 software using 6^th^ order polynomial equations generated from standard curves for interpolation of unknowns.

### Statistical analyses

Post hoc power analysis was performed for all experiments and significance was only considered when power ≥ 0.8. Normality was determined by the Shapiro–Wilk test or Kolmogorov–Smirnov test. For multiple comparisons one-way analysis of variance (ANOVA) or non-parametric (Kruskal–Wallis) tests were performed where appropriate. Reported *P* values were corrected for multiple comparisons (Holm–Sidak correction for ANOVA; Dunn’s correction for Kruskal–Wallis). Unpaired two-tailed Student’s *t*-tests were used for comparisons made between two groups. Curran-Everett guidelines were followed [60].

## Results

### Increased ISG15 expression and ISGylation in cortex, retina, and spinal cord during demyelination

**Fig. 1:**
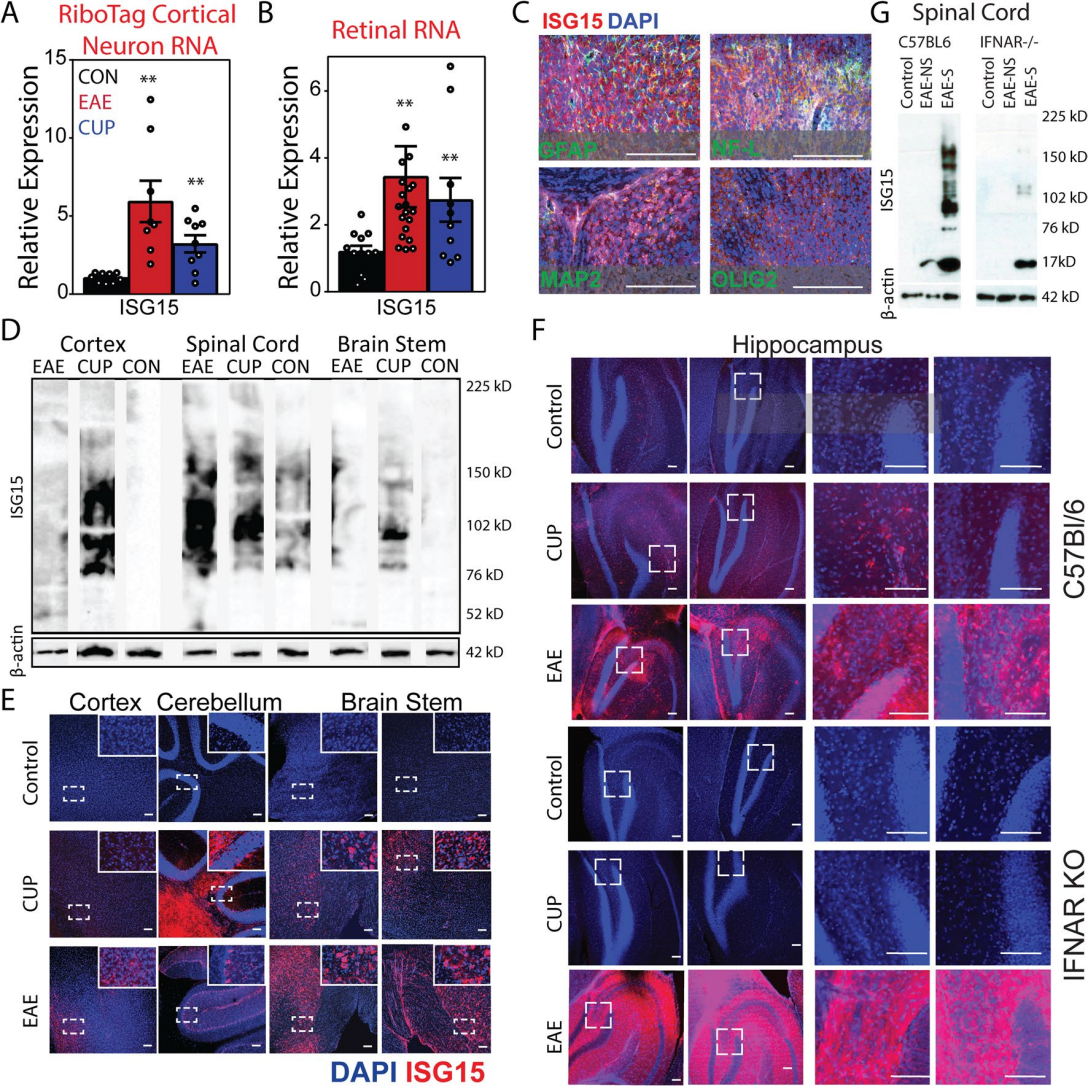
Neuronal upregulation of ISG15 in vivo in demyelinating disease models. ISG15 expression in Syn.Cre-Rpl22 “ribotag” mouse cortex (**A**) or B6 retina (**B**) during EAE (Day 18) or cuprizone (6 weeks) induced demyelination compared to controls. Mean ± SEM are shown. **C** Coronal sections were stained for ISG15 and markers of neuronal dendrites (MAP2) and axons (NFL) as well as glial markers. ISG15 primarily localized to neuronal soma and dendrites. **D** ISG15 immunoblot in cerebral cortex, spinal cord, and brain stem of EAE and cuprizone mice compared to controls showing typical smear pattern indicative of ISGylated targets. **E** Thick-cut vibratome sagittal sections from B6 mice on cuprizone diet, at peak EAE, or controls were immunostained for ISG15 and counterstained with DAPI. Representative confocal images of cortex, cerebellum, and brain stem are shown. **F** Similar ISG15 staining in hippocampus for B6 and IFNAR KO mice on cuprizone diet, at peak EAE, or controls is shown. Scale bars 50 microns. **G** ISG15 immunoblot in spinal cord of non-symptomatic EAE mice (EAE-NS) and symptomatic EAE mice (EAE-S) compared to controls as in **D** for B6 and IFNAR KO mice. **P < 0.05

To verify that ISG15 expression in neurons was upregulated during EAE and cuprizone intoxication we isolated neuronal ribosome-associated transcripts from cortex of mice at peak EAE (day 18) or 6 weeks of cuprizone diet, relative to age matched controls. As shown (Fig. 1a), we measured increased ISG15 expression in cortical neurons of EAE and cuprizone mice compared to controls by RT-PCR. The induction of ISG15 in cortical neurons during cuprizone intoxication induced demyelination suggested that this pathway might be induced in neurons by signals independent of the inflammatory process seen in EAE. Though it should be noted that others have noted immune cell infiltration and IFNg production in cuprizone-induced demyelination [61]. We similarly saw increased ISG15 expression among transcripts isolated from retina at the same time points (Fig. 1b). Notably axons in the optic nerve are all afferent fibers derived from retinal ganglion cells that signal to the lateral geniculate nucleus (LGN). More importantly, the retina is unique in that there are no efferent fibers that signal to the retina. Therefore, unlike most white matter tracks demyelination of the optic nerve may induce deafferentation of LGN neurons but does not induce deafferentation of retinal ganglion cells. This contrasts with the corpus callosum, where demyelination may induce deafferentation of neurons in both hemispheres. Additionally, retinal ganglion cell fibers remain unmyelinated within the eye. Therefore, our observations in the retina indicated that ISG15 induction was not dependent upon anterograde axon intrinsic signals, deafferentation, or local gray matter demyelination. This strongly suggested that ISG15 induction was dependent upon retrograde signals arising from the demyelinated axon. Upon confocal imaging, expression of ISG15 was found to colocalize primarily with MAP2 indicating somatodendritic neuronal expression with limited colocalization with neurofilament light (NFL) positive axons. ISG15 expression was not strongly detected in GFAP + astrocytes or Olig2 + oligodendrocytes or progenitors (Fig. 1c). We also found increased levels of ISGylated proteins in mouse brain and spinal cord by immunoblotting tissue lysates for ISG15 (Fig. 1d). This was associated with increases in ISG15 expression in cortex during EAE compared to control mice. Even more pronounced increases in ISG15 expression were noted in cerebellum, brain stem, and hippocampus in both cuprizone and EAE mice compared to controls (Fig. 1e, f).

### Compared to B6 mice IFNAR KO mice exhibit less ISGylation in CNS during EAE or cuprizone intoxication

ISG15 expression is known to be induced by Type I and Type II interferons. Type I interferons (IFNα isoforms and IFNβ) signal by causing dimerization of the IFNAR1/IFNAR2 subunits of the IFNα receptor (IFNAR). Therefore, we sought to determine whether ISG15 expression was dependent upon IFNAR signaling. We thus induced demyelination by EAE and cuprizone intoxication in IFNAR1 KO. Surprisingly, despite no difference in EAE clinical score in IFNAR KO mice compared to B6 controls (Additional file 2: Fig. S1), we found overall diminished levels of ISGylation in spinal cord (Fig. 1G; WB) and ISG15 expression in CNS tissue of IFNAR KO EAE mice. Comparable results were observed in IFNAR KO mice on cuprizone diet (data not shown). This was contrasted only in hippocampus, where we saw increased levels of ISGylation in IFNAR KO mice during EAE and diminished levels of ISGylation in IFNAR KO mice during cuprizone compared to B6 mice in the same conditions (Fig. 1f). Together these data suggested that while IFNAR signaling may contribute to neuronal ISGylation in cortex and spinal cord it is not required in hippocampus during EAE. This also suggested that EAE clinical onset is not impacted by the absence of spinal cord ISG15 expression and therefore ISGylation is not required for outside-in driven CNS inflammatory disease in WM lesions against myelin peptides. However, given that EAE clinical score is not reflective of underlying gray matter pathology but of spinal cord inflammatory disease, wed did not take this as evidence that ISGylation was uninvolved with GM disease.

### *Axonal treatment with IFNγ but not IFN*α *causes upregulation of ISG15 and increased ISGylation in neurons*

**Fig. 2:**
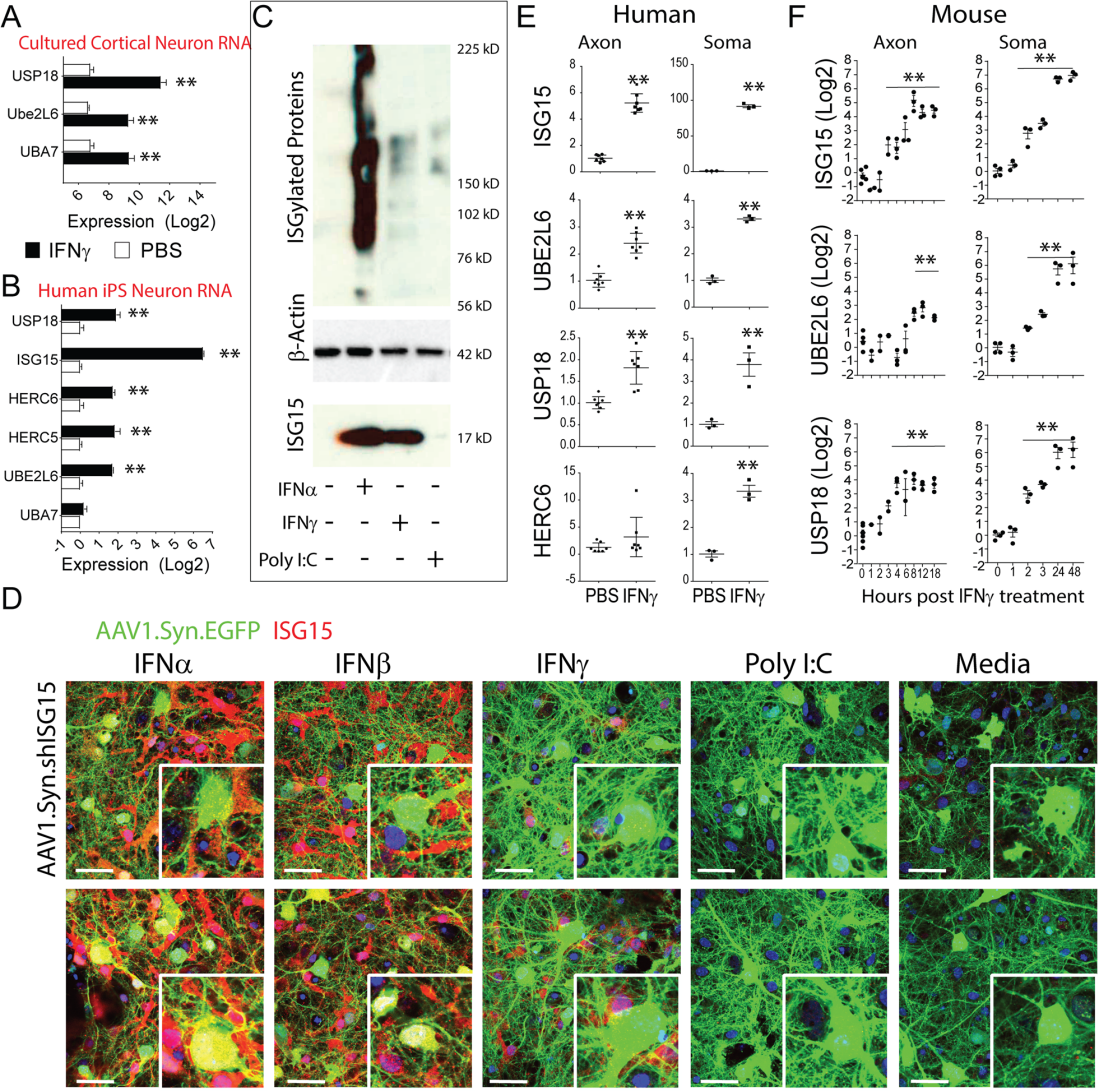
Neuronal upregulation of ISG15 in vitro in response to interferon treatment. Cortical neurons prepared from E15.5 embryonic mouse cortices (**A**) and human IPSC-derived neurons (**B**) were plated into microfluidic axon isolation chambers. After 10 days, axons exhibited dense outgrowth into the distal chamber. At DIV 12, 100 ng/mL IFN*γ* or PBS was added to the distal chamber, and 72 h following axonal treatment with IFN*γ* or PBS gene expression was assessed by microarray. **C** At DIV 12, neurons were treated for 24 h with 2000 U/mL IFNα, 100 ng/mL IFN*γ* or 2 ug/mL PolyI:C to drive ISG15 expression. Lysates were probed for ISG15 (F9; SantaCruz) and acquired on film with Xomat film processor. Membranes were stripped and reprobed for B-actin as loading control. Blots show high molecular weight smear indicative of ISGylated targets and bands at 17kd indicative of ISG15 monomer. Murine cortical neurons (**D**) were transfected with AAV1.Syn.EGFP with or without co-transfection with AAV1.Syn.shISG15 to silence neuronal ISG15 expression and 12 days later were treated for 24 h as in panel C and then fixed and immunostained for ISG15. Representative confocal images show ISG15 expression (red) in IFN-treated neurons (green) as well as non-neuronal cells. Neuronal ISG15 expression was silenced by AAV1.Syn.shISG15 co-transfection. Human IPSC-derived neuronal aggregates (**E**) and murine cortical neurons cultures (**F**) were cultured in microfluidic chambers for 12 days and then IFN*γ* (100 ng/mL) was added to either the axon or soma compartment of the microfluidic chamber. RNA was collected 1–48 h later as indicated and expression levels of the indicated genes were determined by RT-PCR relative to control neurons. Mean ± SEM are shown

Microarray analysis of transcripts induced by IFN*γ* treatment of mouse cortical neurons (Fig. 2a) or human IPSC-derived neurons (Fig. 2b) revealed upregulation of ISGylation pathway genes when the cell soma were treated. This was consistent with prior reports showing that in addition to IFNα and IFNβ, IFNγ could also drive ISG15 expression in immortalized cell lines and leukocytes [62, 63]. To further explore upstream signals of neuronal ISG15 expression and ISGylation we treated mouse cortical neurons or human IPSC-derived neuronal cultures with IFN*γ*, IFNα, IFNβ, and the TLR3 ligand polyinosinic:polycytidylic acid (poly I:C). TLR3 signaling is known to induce IFNα and IFNβ expression in some cell types. Type I and Type II IFNs but not Poly I:C caused increased neuronal ISGylation and increased expression of ISG15 at 24 h post treatment (Fig. 2c, d). Similar results were obtained in human IPSC-derived neurons (Additional file 3: Fig. 3). To determine whether either of these factors was capable of inducing ISG15 expression in neurons through retrograde signals, we infected cortical neurons with an adeno-associated virus to label neurons with green fluorescent protein (AAV1.Syn.eGFP) and cultured these cells in microisolation chambers, allowing neurons to elaborate dense axonal fields into the distal chamber over 12 days. We then treated the axon fields with 2000 U/mL IFNα or 100 ng/mL IFN*γ*. After 24 h cultures were fixed and chambers deconstructed for immunostaining, which revealed that surprisingly axonal treatment with IFN*γ* but not IFNα elicited neuronal ISG15 expression (Additional file 4: Fig S3). Comparable results were found in human IPSC-derived neurons (Fig. 2e; RT-PCR). This suggested that while IFNα may signal on the somatic dendritic armors to induce ISG15 it was not capable of signaling retrogradely at least in our culture platform. In contrast, IFN*γ* signaling was capable of eliciting retrograde signals that led to the induction of ISG15. In a time-course comparison of axonal and somatic treatment we found that ISGylation pathway genes were induced as soon as 2 h after treating cell soma with IFN*γ* and as soon as 4 h after treatment of axons with IFN*γ* (Fig. 2f; RT-PCR).

**Fig. 3:**
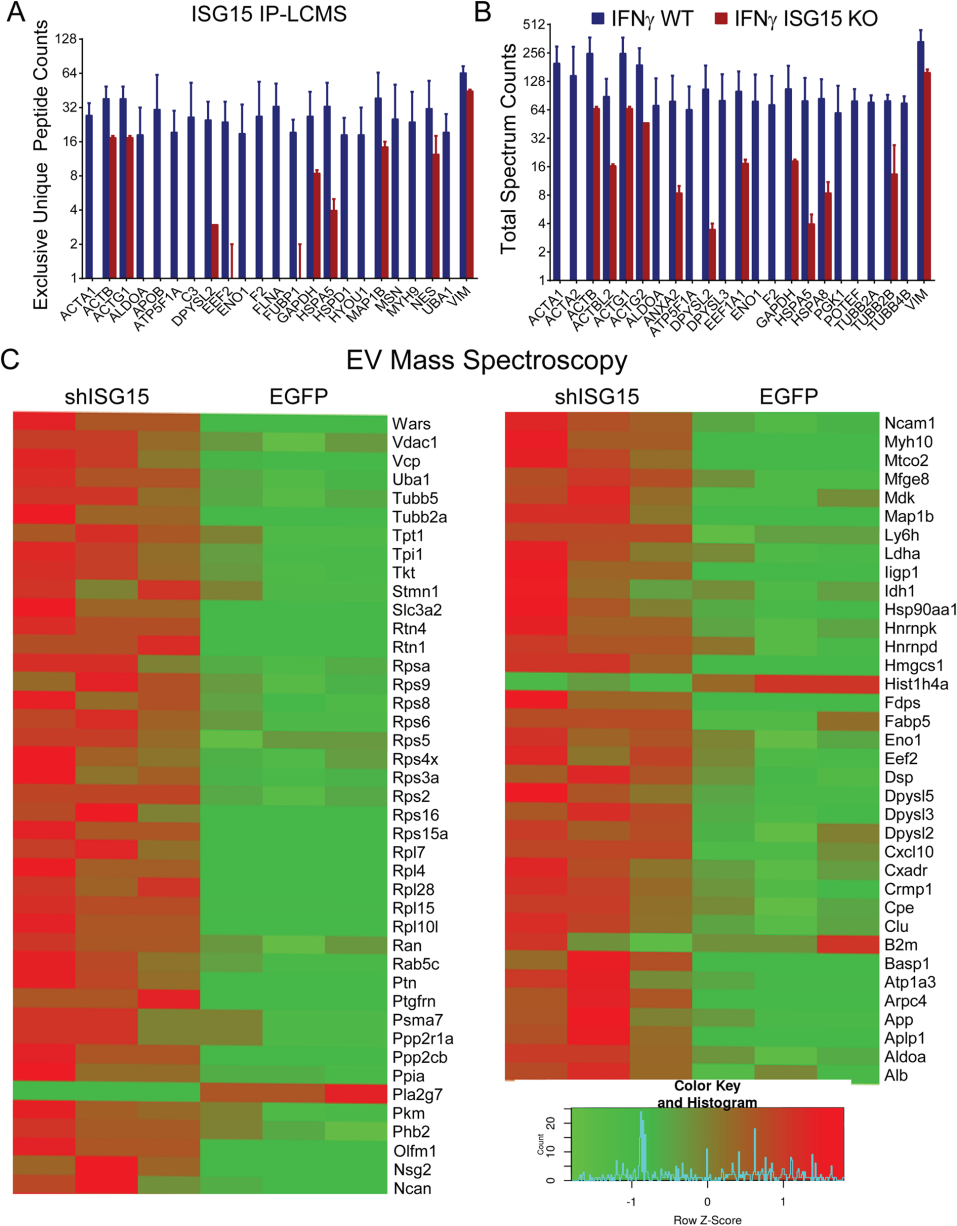
ISG15 induction causes changes in neuronal EV protein content. **A**, **B** Mature cultures of human ISG15 targeted gene knockout or non-targeted control (NTC) neuronal cultures were treated with 100 ng/mL IFN*γ* for 24 h and then lysates were immunoprecipitated with anti-ISG15 (clone F9) and proteins were eluted in 5% glacial acetic acid for washing and LCMS analysis. Proteins with > 5 exclusive unique peptide counts (**A**) or total spectrum counts (**B**) and >5-fold enrichment in NTC precipitates compared to ISG15 KO precipitates were considered significant. Means and SEM are presented. **C** Primary mouse cortical neurons were transfected at DIV 0 with 2000 MOI of AAV1.Syn.shISG15-eGFP or AAV1.Syn.eGFP empty vector control. Media was fully replaced on DIV 7 and cells were treated every other day with an additional bolus of 100 ng/mL IFN*γ*, and EVs were isolated from supernatant 7 days later (DIV 14). Proteins were extracted from exosomal lysates and acquired by LCMS. Proteins with > 5 exclusive unique peptide counts (**A**) or total spectrum counts (**B**) and >5-fold enrichment in shISG15 transfected lysates compared to eGFP transfected controls were considered significant and exclusive unique peptide counts are presented as heat maps. Mean ± SEM are shown

### Identification of ISG15 targets in human neurons

To determine the targets of ISGylation in human neurons, we performed CRISPR/Cas9 gene editing on IPSC-derived human neural stem cells and achieved > 98% knock out of ISG15 in these cells by DNA sequencing, protein ELISA, and RT-PCR-based transcript analysis (Additional file 5: Fig. S4a–e). We then differentiated these cells into neurons alongside non-targeted controls (NTCs). We treated the neurons for 24 h with 100 ng/mL IFN*γ*, collected lysates and immunoprecipitated ISGylated proteins with a monoclonal antibody against human ISG15. We then performed liquid chromatography–mass spectrometry (LC–MS) to identify proteins that were enriched in the NTC neurons relative to those that underwent ISG15 knockdown. Given evidence that ISGylation extends protein half-life by restricting proteasomal targeting, we expected that even after several weeks in culture residual ISG15-conjugated proteins may be present in ISG15 knock down neurons, albeit at lower levels (as evidenced by ELISA). As expected, we found that ISG15 immunoprecipitates from NTC human neurons had more unique protein counts (Fig. 3a) and total spectrum counts (Fig. 3b) than those from ISG15-targeted gene knockout neurons. These proteins included glycolytic pathway proteins (GAPDH, aldolase, enolase, PGK1), cytoskeletal and scaffolding proteins (actin and tubulin subunits, annexin A2, vimentin, filamin, nestin, moesin, myosin 9 and POTEF); CRMP-family proteins involved with axon guidance (DPYSL2/ CRMP2, DPYSL3/CRMP4); and proteins involved with translation and protein folding and the ER stress response (EEF1A1,EEF2, HSPA5, HSPA8, HSPD1, HYOU1, UBA1). We also observed enrichment for synaptic plasticity-associated protein MAP1b, which is known to be enriched in extracellular vehicles (EVs) containing activity associated miRNA. Likewise, many cytoskeletal proteins and glycolytic proteins are known to be packaged into microvesicles and annexin A2 has been recently described as a surface marker for classical microvesicles [64]—raising the possibility that some of these proteins were captured due to incomplete lysis of extracellular vesicles with ISGylated surface proteins.

### ISG15 knockdown alters protein composition of neuronal EVs

**Fig. 4:**
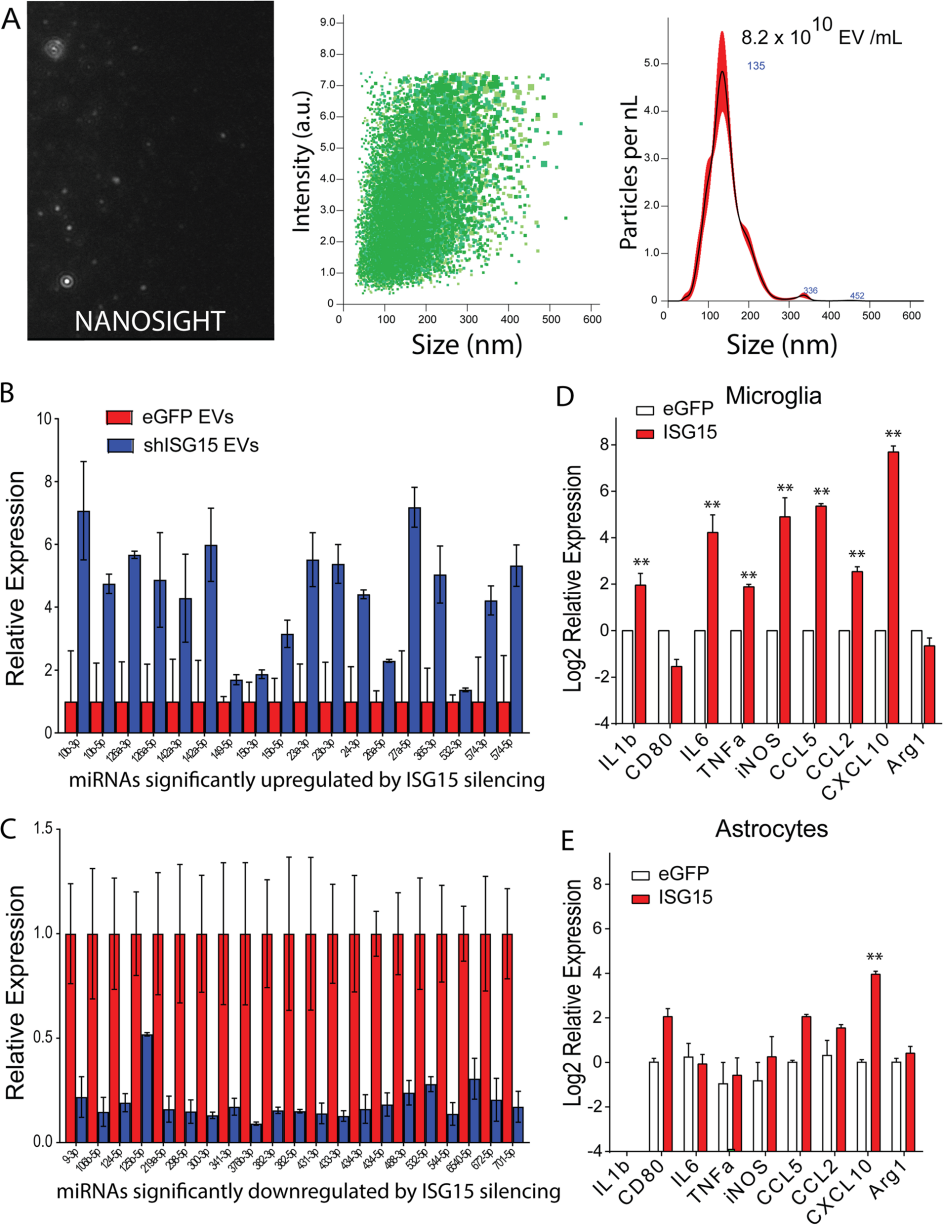
Activation of microglia treated with neuron extracellular vesicles. Extracellular vesicles (EVs) were isolated from primary cortical neurons by centrifugation. To determine EV counts, samples were diluted and analyzed for size and concentration using NanoSight imaging. **A** Representative still frame image from NanoSight acquisition is shown on left. Intensity-size scatter plot and size histogram are shown on right. **B**, **C** Mouse cortical neurons were infected at plating with AAV1.Syn.shISG15 to silence neuronal ISG15 expression or AAV1.Syn.EGFP control and at 12 DIV treated with IFN*γ* for 72 h prior to EV isolation. Micro-RNA was isolated from EVs, and small RNA libraries were prepared and acquired by paired end 100 bp RNA sequencing. Differentially expressed miRNA species were determined using a 5% FDR cut off. MicroRNAs upregulated (**B**) and downregulated (**C**) upon ISG15 knockdown are shown. **D** Primary microglial cultures were prepared from p1 mouse pups. **E** Primary astrocyte cultures were prepared from p3–4 mouse pups. Astrocytes (DIV29) and microglia (DIV7) were treated with EVs from neurons that were infected at plating with AAV1.Syn.ISG15 or AAV1.Syn.EGFP. RNA was isolated from astrocytes and microglia 24 h after EV treatment and expression of the indicated transcripts determined by RT-PCR relative to cells treated for 24 h with control EVs. **P < 0.01 by unpaired Student’s t-test. Mean ± SEM are shown

ISG15 has previously been reported to block EV secretion in some cell types by directly conjugating to TSG101 and promoting lysosomal degradation of multivesiculated bodies [45, 52]. This interferes with secretion of exosomes and arrestin-domain-containing protein 1 (ARRDC1)-mediated microvesicles (ARMMs) as these EVs are known to associate with TSG101 [65, 66]. ARMMs are small (40–100 nm) EVs that were recently shown to bud directly from the plasma membrane. Therefore, we sought to determine how the ISG15 induction following IFN*γ* treatment affects the composition of neuron EVs. We infected murine cortical neurons with AAV1.Syn.shISG15 to ablate neuronal ISG15 expression using a validated protocol for AAV-mediated delivery. Validation of AAV vectors is shown in Additional file 6: Fig. S5. Empty vector controls were used for comparison. After replacing media at day 7 in vitro, we treated cells with IFN*γ* every other day, collected and clarified supernatants 7 days later, and isolated EVs by ultracentrifugation. EVs were characterized by Nanosight (Fig. 4a) as well as NanoFCM (not shown). In separate experiments, we analyzed EV protein content by LCMS. As shown, EVs from AAV1.Syn.shISG15 infected cortical neurons had significantly more exclusive unique peptide counts for several proteins consistent with abrogation of ISG15-mediated blockade of EV secretion. These included proteins involved with vesicle and EV trafficking, cytoskeletal dynamics and synaptic plasticity, regulation of neurite outgrowth and neurotoxicity, proteostasis and stress responses, as well as prominent cytosolic enzymes involved glycolysis (Fig. 3c). Several RNA-binding and ribosomal proteins were similarly enriched in AAV1.Syn.shISG15 EVs. Since EV protein content in ISG15 ablated neurons is determined by both protein sorting as well as diffusion of cytosolic proteins into EVs, it is important to note that changes in EV composition in ISG15 knockdown neurons could be due to ISG15-dependent changes in nuclear import/export, translation, protein localization, or protein degradation. This is especially important to consider for proteins that are principally localized to non-cytosolic compartments under resting conditions such as the ER, nucleus, or nucleolus, where the packaging of these proteins into EVs would be limited. Cytosolic proteins such as ribosomes, cytoskeletal components, and glycolytic proteins are known to be preferentially packaged in EVs that bud of from the plasma membrane including classical microvesicles and ARMMs. This suggested that ISGylation could be selectively disrupting the secretion of these EVs while leaving other EVs unaffected. Indeed, PLA2g7—a phospholipase known to be associated with LDLs—showed increased abundance upon ISG15 knockdown as did histone H4, which is known to selectively associate with amphisomes and dsDNA clearance from cytosol [64, 67, 68]. Meanwhile the B2M-MHC I complex—known to be primarily found on exosomes was unchanged. Again, this suggested that, in contrast to recent reports in other cell types, the secretion of LDLs, amphisomes, and exosomes was not dominantly affected by ISGylation in neurons.

### ISG15 knockdown alters miRNA cargo of EVs from IFNγ-treated neurons

In parallel studies, we sought to investigate how ISG15 affects the miRNA composition of EVs secreted from IFN*γ*-treated neurons. We thus performed RNAseq analysis of EV-associated miRNA transcripts isolated from IFN*γ*-treated murine cortical neurons as described above. By this approach we identified transcripts that were upregulated or downregulated in AAV1.syn.shISG15 transduced neurons relative to AAV1.Syn.eGFP transduced controls. Ablation of ISG15 in neurons caused differential expression of several EV-associated miRNA transcripts. Intriguingly we saw increased levels of miRNAs that have been shown to promote quiescence or suppress cytokine secretion in macrophages/microglia, astrocytes, or endothelial cells (Fig. 4b). These included miR-15b [69]; miR-23a/b and miR-27a [70–73]; miR-126a [74–76]; miR24, miR-26a, miR-149 [77–84]; and miR-365 [85]. Additionally, several of these upregulated mi-RNA transcripts have also been shown to be neuroprotective including miRNA 23a, miR-23b, miR-24, and miR-27a [86–101]. Finally, EVs from ISG15 knockdown neurons exhibited downregulation of several mi-RNA transcripts that have been shown to promote microglial or macrophage activation and cytokine secretion or neuronal toxicity (Fig. 4c). These included miR-9, miR-106b, miR-125b, miR-298, and miR-431 [85, 102–118]. Together, these data indicated that ISG15- caused neurons to skew expression of EV-associated miRNAs toward proinflammatory miRNA and neurotoxic miRNAs and away from anti-inflammatory and neuroprotective miRNAs. See discussion for further analysis.

### EVs from neurons expressing ISG15 cause microglia to upregulate expression of inflammatory cytokines

Importantly, neurons are known to communicate through EVs to microglia and astrocytes to maintain quiescence during the steady state [75, 82, 119–133]. Transfer to microglia is thought to promote clearance of aggregated proteins from stressed neurons and in some cases may contribute to the eventual activation of microglial toward a neurodegenerative phenotype. Microglia that acquire a neurodegenerative phenotype are thought to be more proinflammatory and may also contribute to disease spread through secondary secretion of aggregate laden EVs that are taken up by healthy neurons [134, 135]. Given that neurodegeneration and gliosis are features of GM pathology in MS, we sought to determine whether neuronal ISG15 expression contributed to microglial or astrocyte expression of proinflammatory cytokines by altering neuronal EV composition. We infected mouse cortical neurons with an adeno-associated virus to drive overexpression of ISG15 (AAV1.Syn.ISG15) or empty vector control (AAV1.Syn.EGFP) and isolated neuronal EVs from clarified supernatants at 12 days in vitro. We then treated mature primary mouse microglia and mixed astrocyte cultures with these EVs and measured expression of inflammatory transcripts by RT-PCR 24 h later. As shown, (Fig. 4d) EVs from ISG15-overexpressing neuronal cultures caused microglia to increase expression of IL1b, IL6, TNFa, iNOS, CCL5, CCL2, and CXCL10 compared to mock infected neuronal EVs. Similar effects were observed to a lesser extent in mixed glial cultures, which comprised primarily astrocytes with a less abundant population of microglia (Fig. 4e). This suggested that astrocytes were not primarily responsible for the observed effects. We also noted that microglia treated with EVs from neurons overexpressing ISG15 were more likely to exhibit a dendriform morphology and less likely to exhibit a bipolar morphology than microglia treated with PBS or EVs from mock infected neurons and exhibited reduced expression of TWEAK (data not shown). TWEAK through interaction with its receptor Fn14 has previously been reported to be involved in microglia-dependent synaptic remodeling [136, 137].

### Soluble ISG15 causes increased expression and secretion of inflammatory cytokines by microglia

**Fig. 5:**
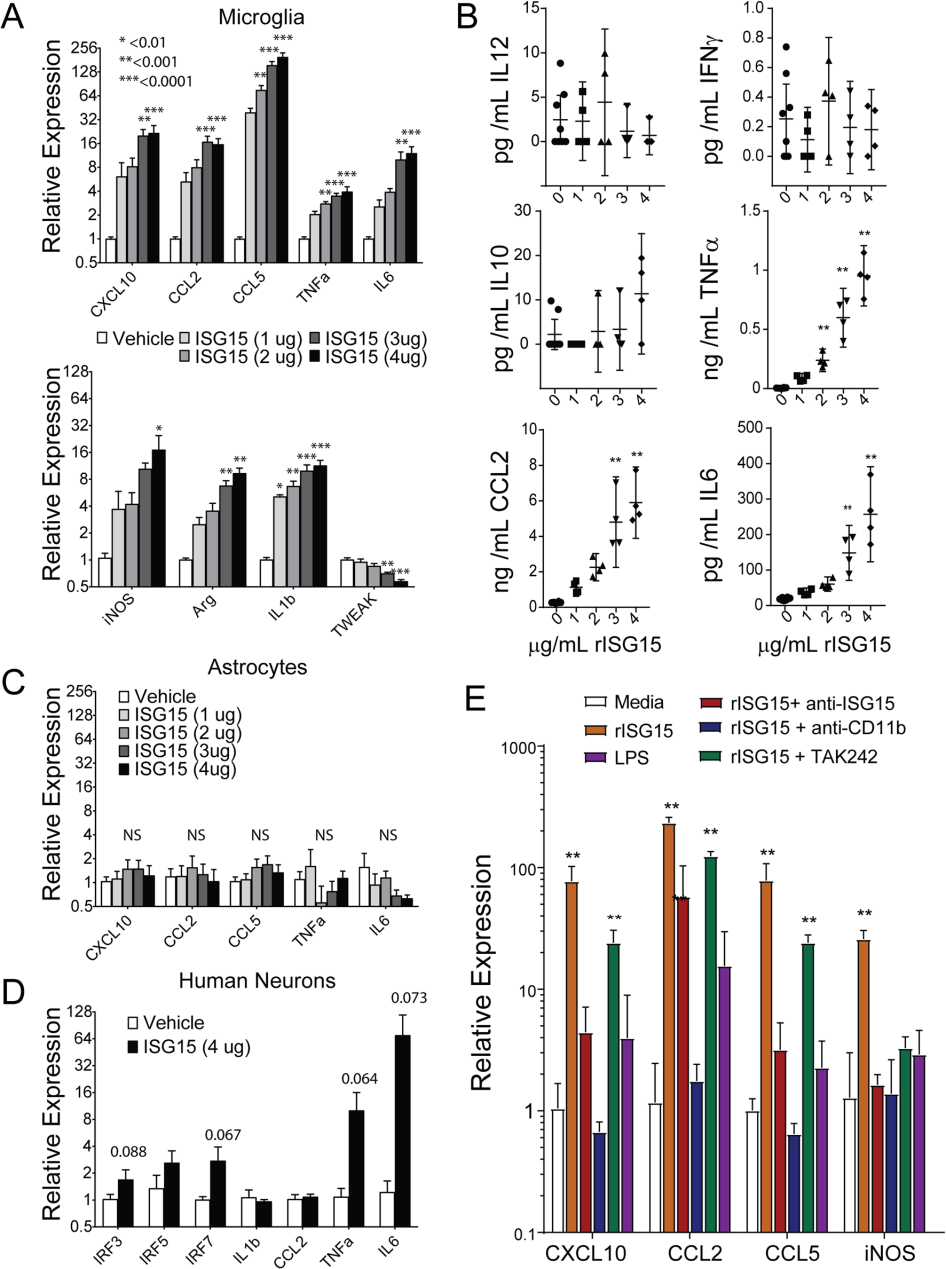
Activation of microglia treated with ISG15. Primary microglial cultures were prepared from p1 mouse pups. Primary astrocyte cultures were prepared from p3-4 mouse pups. Human IPSC-derived neurons were differentiated from NSC in media containing cAMP, BDNF, IGF1, GDNF, and StemPro neurosupplement for > 2 weeks. Astrocytes (DIV29) and microglia (DIV7) were treated with ISG15 at the indicated dose. Chemokine and cytokine mRNA (**A**) and protein (**B**) expression 24 h following treatment of murine microglia with ISG15 as assessed by RT-PCR and cytometric bead array, respectively. Relative expression of mRNA for cytokines, chemokines, and transcription factors in murine astrocytes (**C**) or IPSC-derived human neurons (**D**) 24 h following treatment with ISG15 as assessed by RT-PCR. **E** Relative expression of activation markers in microglia treated as in panels A and B following co-treatment with neutralizing antibodies against ISG15 or CD11b, co-treatment with the TLR4 inhibitor TAK242, or treatment with LPS. *P *values are indicated for one-way ANOVA. *P < 0.01, **P < 0.001, ***P < 0.0001. Mean ± SEM are shown

We observed that treatment with 100 ng/mL IFN*γ* caused increased levels of soluble ISG15 in cell supernatants from cultured neurons. Of note ISG15 secretion could be restored in ISG15 deficient neural cultures where ISG15 was selectively restored in neurons using AAV1.Syn.ISG15 transduction (Additional file 5: Fig. S4e, f). Prior reports have shown that secreted ISG15 can drive increased IFN*γ* secretion by T cells, increased proliferation and cytolytic activity of NK cells, as well as increased recruitment and activation of myeloid cells [138–148]. It was later shown that in T cells and NK cells activation by dimerized ISG15 is mediated by binding with surface CD11a [149, 150]. To determine the relevance of neuron secreted ISG15 on glial activation we treated murine microglial and astrocyte cultures as well as human mixed neural cultures containing neurons, oligodendrocytes, and astrocytes with soluble ISG15 for 24 h and measured cytokine gene expression by RT-PCR and secretion by cytometric bead array. In ISG15 treated microglial cultures, we observed a dose dependent increase in cytokine expression (Fig. 5a) and secretion (Fig. 5b) as well as a dose dependent decrease in TWEAK mRNA expression (Fig. 5a). In contrast, at 24 h rISG15 had negligible effect on astrocytes and no significant effects on mixed human neural cell cultures (Fig. 5c, d). Although CD11a/CD18; αLβ2 integrin has been reported as a receptor for ISG15 in NK cells and T cells, microglia do not express this receptor. They do express high levels of the structurally related CD11b/CD18, αMβ2. Importantly, these genes are thought to be the result of gene duplication events and are structurally related (REF). For example, both of these integrins have been shown to bind to ICAM1 albeit with differing affinities (REF). This indicated the possibility that CD11a and CD11b may share other ligands. Therefore, we hypothesized that ISG15 might be an additional shared ligand capable of binding CD11b. In line with this hypothesis, we found that microglial activation by rISG15 was blocked by neutralizing antibodies against CD11b (Fig. 5e).

### ISG15 is upregulated in neurons in MS perilesional normal-appearing GM

**Fig. 6:**
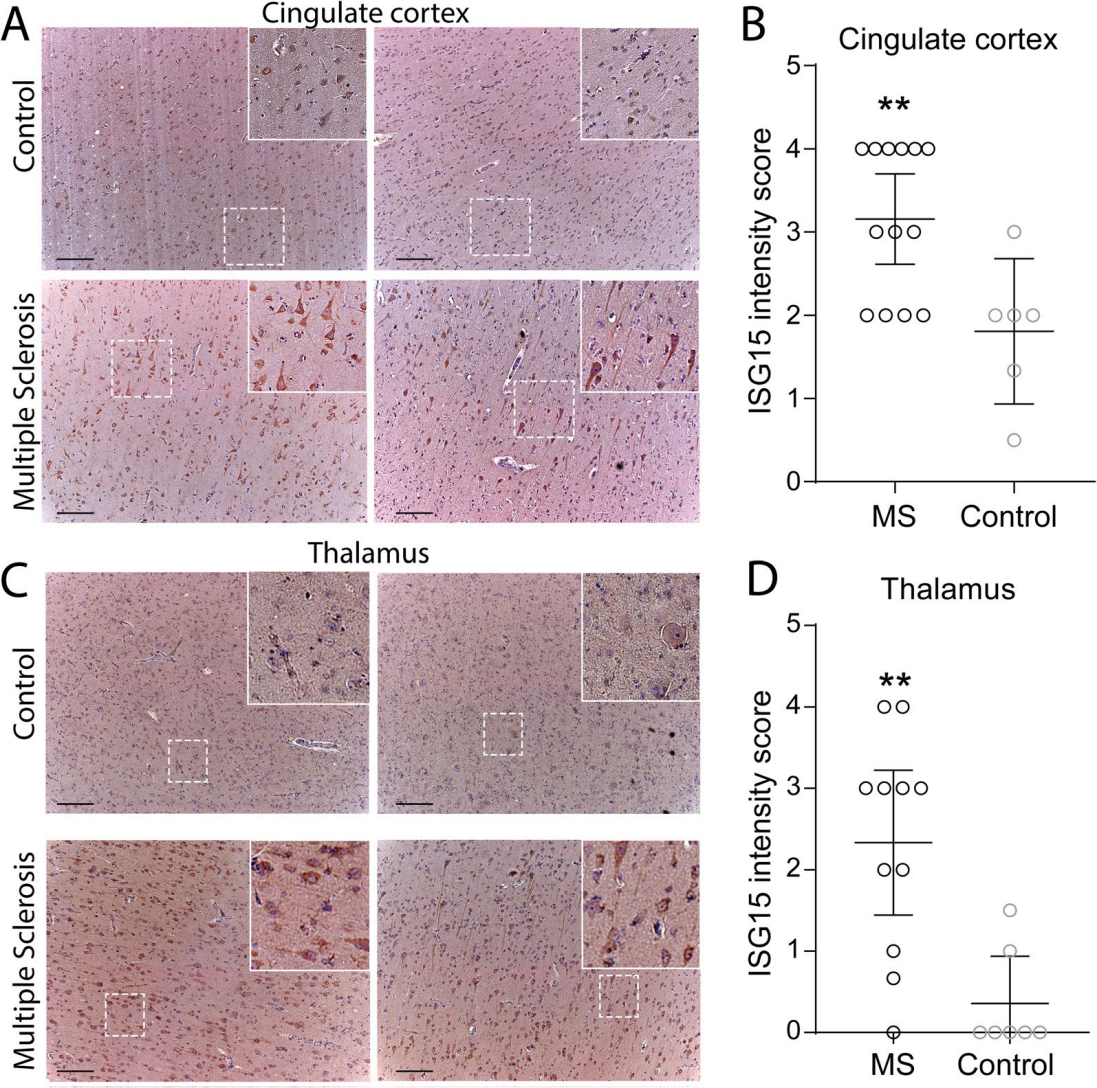
Cortical and deep gray matter in MS exhibit neuronal ISGylation. Paraffin-embedded MS postmortem brain tissue was obtained from Mayo Clinic tissue registry, Netherlands Brain bank, and Normal Aging Brain Collection Amsterdam (controls). Tissues were deparaffinized and antigen retrieved in 10 mM Tris 1 mM EDTA pH9.0 for 20 min at 95 C. Neuronal ISG15 immunostaining (brown) in cortical gray matter (**A**, **B**) and deep gray matter (**C**, **D**) from normal aged controls and MS patients. Representative micrographs shown in **A** and **B**. Qualitative staining scores are shown in **B** and **D**. Tissues were counterstained with hematoxylin (blue). Representative micrographs are shown. Insets are digitally magnified. **P < 0.01 by unpaired Student’s t-test. Scale bar = 100 microns. Mean ± SEM are shown

To determine the disease relevance of neuronal ISG15 expression, we performed immunostaining on 46 MS brain tissue blocks as well as non-MS control sections from 20 brain tissue blocks (Additional file 1: Table S2). We found evidence of increased ISG15 expression in neurons in MS cingulate cortex (MS = 3.154 ± 0.249; Control = 1.806 ± 0.340) and in thalamic nuclei (MS = 2.333 ± 0.399; Control = 0.357 ± 0.237) compared to controls (Fig. 6a–d). ISG15 expression was observed in neurons in normal-appearing cortical tissue and in tissues with subpial, intracortical or juxtacortical lesions. Astroglial expression of ISG15 was observed in white matter tissues, especially within and adjacent to demyelinating plaques (data not shown). We also found evidence of basal expression of ISG15 in Purkinje neurons in cerebellum that was unchanged in MS (MS = 4.776 ± 0.113; Control = 4.607 ± 0.393; Additional file 7: Fig. S6a, b); however, rare ISG15 + axon fibers within cerebellar white matter tracts were exclusively found in MS patients (arrows Additional file 7: Fig. S6b). These data provide evidence of increased neuronal ISG15 expression in MS patient gray matter. Follow-up, in situ hybridization studies in patient tissues (Additional file 2: Table S2) failed to demonstrate a significant difference in expression of the ISG15 E3 ligase HERC5 in cingulate cortex from MS patients compared to controls (MS = 0.1348 ± 0.0390; Control = 0.2428 ± 0.0740)—though a non-significant trend toward decreased expression of the ISG15 peptidase USP18 was noted (MS = 0.6811 ± 0.0977; Control = 1.2850 ± 0.3414; Additional file 8: Fig. S7). Similarly, we found no significant differences in mRNA expression for these genes in temporal cortex tissues from patients with Alzheimer’s disease compared to controls (AD HERC5 = 0.1393 ± 0.0357; Control HERC5 = 0.0917 ± 0.0246; AD USP18 = 1.116 ± 0.4015; Control USP18 = 0.6479 ± 0.2029; data not shown).

## Discussion

Prior reports have noted ISG15 upregulation in models of focal and global cerebral ischemia, CNS viral infection, TBI associated ALS, ataxia-telangiectasia, and to a limited extent in viral and toxic models of demyelinating disease [39, 40, 47, 151–156]. Yet, this is the first report to our knowledge to extensively focus on the role of neuronal ISGylation induced in MS. Importantly, we report evidence that secretion of ISG15 dimers by IFN*γ*-treated neurons might promote microglial activation. Secreted ISG15 homodimers have been shown to promote IFN*γ* secretion from T cells and promote activation of myeloid cells [138–146, 148–150]. Free ISG15 has recently been shown to be an alarmin that enhances cytotoxic responses from CD8 + T cells and NK cells by binding to CD11a [147]. We have shown here that ISG15 similarly can promote production of chemokines, inflammatory cytokines, and iNOS from microglia—an effect that seems to be at least partially dependent upon CD11b. Furthermore, we provide evidence that cultured neurons can secrete ISG15 and that this secretion is upregulated by IFN*γ* treatment. Since ISG15 dimerization and secretion is favored in the absence of nitric oxide (NO) production, this may well represent one of the earliest impacts of neuronal ISG15 expression. Increased microglial iNOS expression in response to ISG15 secreted from neighboring neurons would lead to elevated levels of NO. NO could then freely diffuse across cell membranes and cause nitrosylation of cytosolic ISG15 being synthesized in neighboring neurons. Cysteine nitrosylation is known to inhibit ISG15 dimer formation and promote ISGylation [157]. This increased ISGylation might then skew the miRNA composition of neuronal EVs to promote further microglial activation. Specifically, we found that IFN*γ* treatment caused ISG15-dependent upregulation of proinflammatory and neurotoxic miRNAs (miR-9, miR-106b, miR-125b, miR-298, and miR-431) as well as downregulation of anti-inflammatory and neuroprotective miRNAs (miR-15b, miR-23a/b, miR-27a; miR-126a, miR24, miR-26a, miR-149, and miR-365). These are further discussed below.

### Proinflammatory and neurotoxic EV-miRNAs increased by ISG15-dependent IFNγ signaling in neurons

ISG15 ablation in neurons prevented IFN*γ*-induced elevation of miRNAs thought to promote microglial activation. For example, miR-9 is a neuron specific miRNA that is known to target MCPIP1 to promote microglial cytokine secretion [102]. Both miR-106b and miR298 have been implicated in cerebral ischemia reperfusion injury, with the latter promoting autophagic and apoptotic neuron loss by targeting Act1 [103]. Additionally, miR-106b is upregulated in Alzheimer disease models and has been shown to cause neuronal amyloid-beta accumulation and microglia M1-skewing by targeting ABCA1 and TGFb signaling [104, 105]. Likewise, studies have shown that miR-125b promotes NFkB signaling, TNFa secretion, and an uncontrolled toxic M1 reaction by targeting A20 and STAT3 in microglia [85, 106–116]. MiR-125b and miR-431 have also been implicated in motor neuron death and neurite loss in spinal muscular atrophy [117, 118]. Moreover, many of these miRNAs are also upregulated in brain or spinal cord during disease including miR-341 in chronic constrictive injury model [158], miR-376 in prion disease prodrome [159] and Parkinson’s disease [160], miR-433 in Parkinson’s disease [161], miR-6540 in B6 EAE peak disease [162], and miR-106b in stroke and Alzheimer’s disease models [163–165].

### Anti-inflammatory and neuroprotective EV-miRNAs decreased by ISG15-dependent IFNγ signaling in neurons

Some of the miRNAs elevated in neuronal EVs by ISG15 ablation suppress microglial activation. MiR-149, miR-24 and miR-26a restrict microglial expression of TNFa, IL1b, and COX2—by targeting HMGA2. In line with this, several of these miRNAs—including miRNA-149 miRNA-26a and miRNA-27a—are downregulated in microglia during LPS stimulation and overexpression of these miRNAs has been shown to reduce microglial and macrophage responses to LPS by targeting elements of the TLR and IL1R signaling pathways including TLR4, MyD88 and IRAK4. In addition to effects in glial cells miR-23a, miR-23b, and miR-27a may contribute to CNS immune privilege by promoting maintenance of the blood brain barrier [73, 74, 76, 80, 166, 167]. Additionally, several of these upregulated mi-RNA species have been shown to be neuroprotective. For example, miRNA 23a, miR-23b, miR-24, and miR-27a are known to target APAF1 and pro-apoptotic BCL family proteins and thereby prevent neuronal apoptosis, and downregulation of these miRNAs during acute EAE, TBI, spinal cord injury, and stroke is thought to contribute to neuron and neurite loss [86–98]. Direct protection of neurons has been shown for miR-23a in spinal muscular atrophy [99] and for miR-27a in EAE and TBI by, respectively, reducing excitotoxicity through suppression of glutamate receptor subunits [168] and reducing oxidative injury by suppressing FOXO1 and FOXO3a [100, 101]. Importantly, glutamate receptors are also expressed on endothelial cells, where they promote vascular permeability. Taken together these results suggest that in aggregate the miRNAs that were increased by ISG15 ablation promote neuroprotection.

Further work fully exploring the effects of EV protein and miRNA composition changes induced by ISGylation during MS and neurodegenerative diseases is warranted. For example, it is unclear in the present study the extent to which some of the observed effects are dependent upon ISGylation, ISG15 dimer secretion, heretofore undescribed impacts of free ISG15 accumulation within cellular compartments. Additionally, whether neuronal ISGylation has concomitant protective effect in the CNS is still unknown. For example, elevated ISGylation has previously been reported in a model of transient cerebral ischemia reperfusion injury where it was found to be protective [153, 156]. In this study, nearly 40 proteins were reported to be ISGylated in these tissues, yet they remained mostly unidentified. Here we report a list of putative protein targets in neurons many of which are involved with glycolysis, cytoskeletal structure, axon guidance, as well as protein translation and folding. For instance, several of the several cytoskeletal proteins that were selectively pulled down with anti-ISG15 have been found to be enriched in exosomal cargo. It remains to be determined whether this enrichment reflects direct ISGylation of these targets or ISGylation of surface proteins on incompletely lysed exosomes or other EVs that contain these proteins. Additionally, ISG15 has been previously reported to regulate cytoskeletal dynamics, and as such it is possible that neuronal ISGylation of cytoskeletal proteins could contribute to processes such as interferon-induced dendritic atrophy [169, 170], organelle localization, or other morphological changes. Indeed, since many post synaptic density proteins rely on cytoskeletal proteins and scaffolding proteins to maintain synaptic localization [171–180], ISGylation of these proteins could induce synaptic protein redistribution (e.g., to autophagosomes [51]) leading to neuronal dysfunction. This possibility remains to be formally explored. Likewise, ISGylation of heat shock proteins and elongation factors could limit protein synthesis and disrupt proper folding as described in studies on how ISG15 blocks viral replication [181–184]. Combined with the known role of ISGylation in disrupting ubiquitin-dependent targeting of proteins for proteasomal degradation [39, 40, 44], ISGylation may end up having a profound effect on neuronal proteostasis by restricting both protein synthesis and turnover—an effect that on the one hand could be protective in the acute setting by temporarily placing the neuron in ‘protein stasis’ that has been described for adaptive translational pausing [185] or torpor [152, 156] while on the other hand contributing to neurodegeneration and cognitive impairment in the long-term by limiting the neuronal capacity to degrade misfolded proteins or rapidly respond to external stimuli. Indeed, the question of whether ISGylation inhibits proteasomal degradation in neurons has a great deal of significance for primary neurodegenerative diseases involving protein aggregation.

In summary, therapies that are effective for progressive MS are crucially needed. For that, we first need a better understanding of the discrete mechanisms that drive neuronal dysfunction and diffuse neuron loss following demyelination. We have identified ISGylation as a principal component of the neuronal response to demyelination which—in the chronic setting—may underlie in part the GM pathology seen in MS. Future work will focus on the functional consequences of neuronal ISGylation by determining how this pathway affects protein turnover, glial activation, and mitochondrial dynamics. This signaling axis may constitute a causal link between inflammatory WM lesions and the diffuse GM pathology associated with neuron loss. If true, therapeutic strategies targeting neuronal ISG15, ISG15-specific E3 ligases, or key EV-associated miRNAs that are disrupted by ISG15 induction may prove invaluable to halting GM atrophy-related clinical progression and to mitigating or reversing cognitive impairment in patients with MS.

## Conclusions

**Fig. 7:**
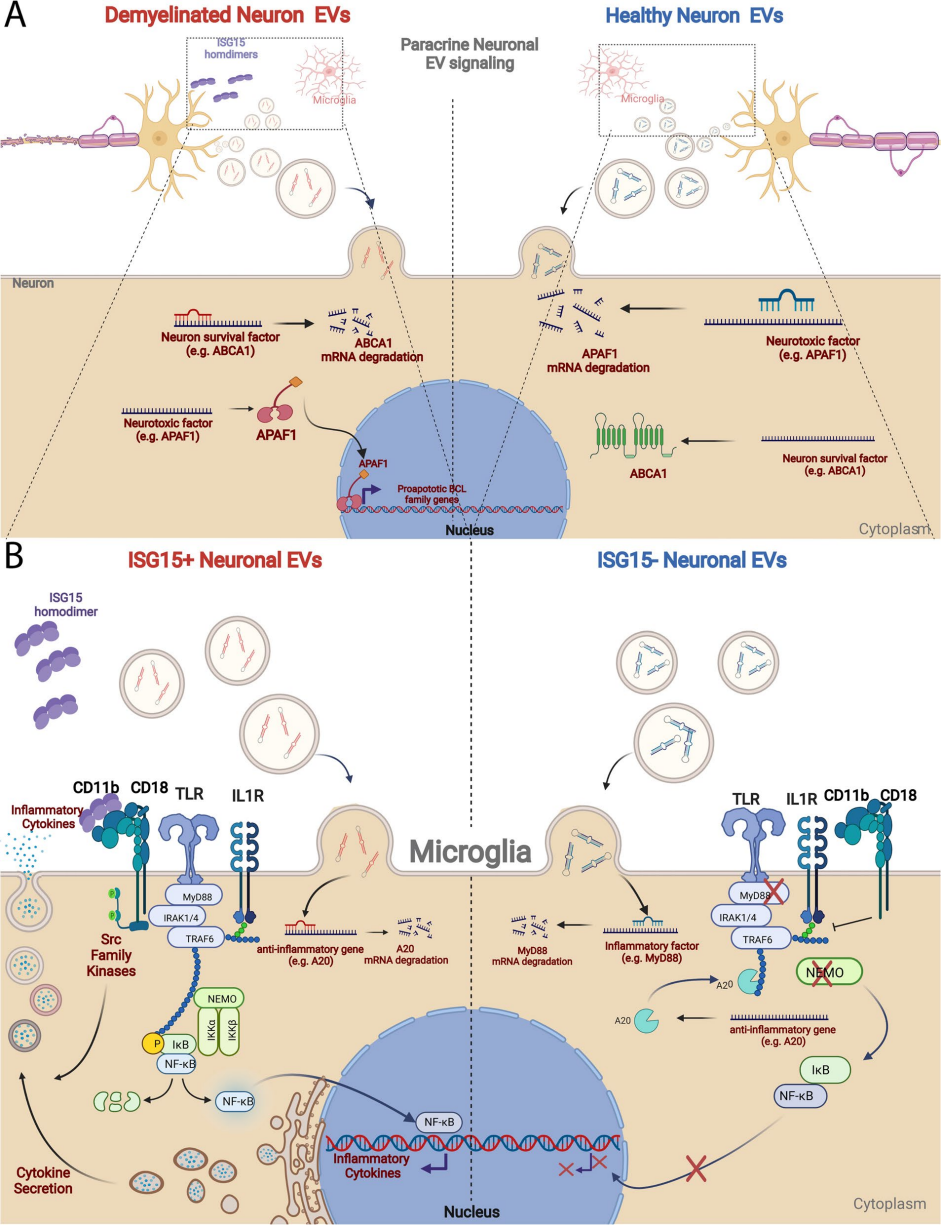
Schematic summary of proposed model. **A** In response to demyelination neurons express and secrete ISG15. Induction of ISGylation also results in altered miRNA composition of secreted EVs. These EVs are taken up by surrounding neurons resulting in increased miRNA targeting of neuroprotective transcripts and reduced miRNA targeting of pro-apoptotic transcripts. **B** ISG15 secreted by neurons binds CD11b causing increased expression and secretion of proinflammatory cytokines. Additionally, EVs from ISG15 + neurons are also taken up by microglia resulting in reduced expression of anti-inflammatory transcripts such as A20

Summary findings and example miRNA targets in neurons and microglia are illustrated in Fig. 7:

- ISG15 is induced in neurons in response to both toxic and inflammatory demyelination.
- Secreted ISG15 acts on microglia to induce expression iNOS and secretion of inflammatory cytokines.
- ISG15 induction and ISGylation in neurons alters the protein and miRNA composition of EVs released by neurons.
- EVs from ISG15 + neuronal cultures support further microglial activation and cytokine secretion.

## Supplementary Information

**Additional file 1: Tables S1 and S2.**

**Additional file 2: Figure S1.** No difference in EAE severity between B6 and IFNAR KO mice. B6 and IFNα receptor knock out (IFNARKO) mice were immunized subcutaneously with 100 ug MOG35-55 and given 200 ng pertussis toxin on the day of immunization and 2 days later to induce experimental autoimmune encephalomyelitis (EAE). Clinical scores (detailed in methods section) were recorded daily beginning 7 days later. Mean clinical scores at 12 days post immunization (early EAE) and 18 days post immunization are plotted for both groups. Mean ± SEM are shown.

**Additional file 3: Figure S2.** ISG15 induction in human neurons by IFN treatment. Human IPSC-derived neurons (E) were transfected with AAV1.Syn.EGFP with or without co-transfection with AAV1.Syn.shISG15 to silence neuronal ISG15 expression and 12 days later were treated for 24 h with 2000 U/mL IFNα, 100 ng/mL IFN*γ* or 2 ug/mL PolyI:C to drive ISG15 expression. Neurons were then fixed and immunostained for ISG15. Representative confocal images show ISG15 expression (red) in IFN-treated neurons (green) as well as non-neuronal cells. Neuronal ISG15 expression was silenced by AAV1.Syn.shISG15 co-transfection.

**Additional file 4: Figure S3.** IFN*γ* but not IFNα causes retrograde induction of ISG15 in cortical neurons. Primary mouse cortical neurons were cultured in microfluidic axon isolation chambers and allowed to elaborate axons into the distal chamber. Neurons were transfected with AAV1.Syn.eGFP, AAV1.Syn.shISG15-eGFP as indicated. At DIV12 axon fields were treated with 2000 U/mL IFNα or 100 ng/mL IFN*γ* for 24–72 h and then cells were fixed and stained for ISG15 (red). DAPI-stained neuronal nuclei are shown in blue. Scale bars 100 microns.

**Additional file 5: Figure S4.** CRISPR/Cas9 gene editing of ISG15 in human IPSC-derived neural stem cells. Neural stem cells from human IPS cells were treated with three guide RNAs targeting ISG15 together with Cas9 complexes. Cells were diluted, plated, and allowed to grow out for 2 + weeks. DNA was then isolated and analyzed by Sanger sequencing. A) A single sequence exhibiting ISG15 gene truncation comprised > 98% of all analyzed sequences. B) Synthego ICE analysis showing guide RNAs and PAM sequences that were used for CRISPR-Cas9 editing along with % indel, model fit, and calculated knockout score. C-E) Following ISG15 knockdown, human IPSC-derived neurons or unedited parental cells were treated with 100 ng/mL IFN*γ* for 24 h as indicated and then we determined ISG15 protein levels in cell lysates (ELISA; C), ISG15 mRNA expression levels (RT-PCR; D), and ISG15 protein concentration present in cell supernatant (ELISA; E). F) We selectively restored ISG15 expression to neurons with AAV1.Syn.ISG15 transfection of ISG15 KO IPSC-derived human neurons and then treated these cells with 100 ng/mL IFN*γ* to measure neuronal ISG15 secretion. G) Discordance of “knockout” Sanger sequencing results with control ISG15 sequence showing expected ~ 25% concordance in target region. H) Sequence of ISG15 edited NSCs shown alongside the sequence from controls NSCs shows that discordance emerges at the guide 2 cut site (~ bp180). Mean ± SEM are shown. *P < 0.01 by unpaired Student’s t-test.

**Additional file 6: Figure S5.** RT-PCR- and immunofluorescence-based validation of adeno-associated viral vectors for driving ISG15 and ISGylation regulatory proteins in neuronal cultures. To validate AAV-mediated expression of ISG15, and USP18, cortical neurons were infected at plating (DIV 0) with 2000 multiplicities of infection (MOI) of AAV1.Syn.eGFP control vector or each of the experimental vectors: AAV1.Syn.ISG15 (A), AAV1.Syn.USP18 (B), or AAV1.Syn.HERC6 (C) and mRNA expression determined by RT-PCR for the indicated conditions. D) The extent of neuronal ISGylation was elevated by AAV1.Syn.ISG15 as determined by immunofluorescent staining for ISG15 in each infection group (co-infected with AAV1.Syn.eGFP to identify neurons) quantified in ImageJ software (E). Murine cortical neurons were infected with AAV1.Syn.shISG15-eGFP or AAV1.Syn.eGFP control vector treated with IFN*γ* or PBS vehicle control. As shown IFN*γ*-treatment induced expression of endogenous ISG15 was incompletely suppressed by shISG15 (G), perhaps due to expression of ISG15 in non-neuronal cells such as astrocytes known to be present in these cultures. H) Neuron cultures infected with both AAV1.Syn.Cre-eGFP and AAV1.EF1alpha.NBL10 (which bear neuron-restricted HA-Tagged ribosomal subunits Rpl10) and co-infected with or without AAV.Syn.shISG15 were treated with IFN*γ* or PBS as indicated. We isolated neuronal ribosome-bound mRNA from these cultures using anti-HA.11 immunoprecipitation and performed RT-PCR to determine neuronal active translation of ISG15 transcripts. In these experiments, we found that neuronal induction of ISG15 translation was completely abrogated by shISG15. I) Cortical neurons were infected and treated as in G and then fixed, permeablized, and stained for ISG15. Images were acquired on an Axioscope. Mean fluorescence intensity of ISG15 stain in eGFP + neurons was determined using Image J macros. J) IPSC-derived human neurons were infected with AAV1.Syn.ISG15 or mock infected and treated at DIV 12 with IFN*γ* or vehicle control. Lysates probed for ISG15 by Simple Western indicated increased ISGylation induced by IFN*γ* treatment that was further enhanced by ISG15 overexpression. ** P < 0.05. Mean ± SEM are shown.

**Additional file 7: Figure S6.** Axonal ISGylation in MS cerebellum. Paraffin-embedded MS postmortem brain tissue was obtained from Mayo Clinic tissue registry, Netherlands Brain bank, and Normal Aging Brain Collection Amsterdam (controls). Tissues were deparaffinized and antigen retrieved in 10 mM Tris 1 mM EDTA pH9.0 for 20 min at 95 C. Immunostaining is shown for ISG15 (brown). Tissues were counterstained with hematoxylin (blue). The extent of neuronal ISGylation was quantified in cerebellum (A). Representative micrographs are shown in B. Insets are digitally magnified. Arrows indicate areas of ISG15 staining on axons in cerebellar white matter tracts. ISG15 staining intensity scores are shown on left. Scale bar = 100 microns. Mean ± SEM are shown.

**Additional file 8: Figure S7.** Top) Signal quantification of in situ hybridization with probes against USP18 and HERC5 in paraffin embedded temporal cortex tissue sections from patients with Alzheimer’s disease (AD; n = 5) and normal controls (NC; n = 4). Bottom) Similar quantification of in situ hybridization in paraffin embedded cingulate cortex tissue sections from patients with multiple sclerosis (MS; n = 4) and normal controls (NC; n = 3)

## Data Availability

Data sharing is not applicable to this article. Please contact the author for data requests.

## Abbreviations

AAV: Adeno associated virus
BDNF: Brain-derived neurotrophic factor
EAE: Experimental autoimmune encephalomyelitis
EV: Extracellular vesicles
GM: Grey matter
IFNAR: Interferon alpha receptor
IGF1: Insulin-like growth factor 1
ISG15: Interferon stimulated gene 15
MS: Multiple sclerosis
NFL: Neurofilament light
NO: Nitric oxide
NTC: Non-targeted control
PDMS: Polydimethylsiloxane
