# Supplementary material for: ISGylation is induced in neurons by demyelination driving ISG15-dependent microglial activation
Source: J Neuroinflammation. 2022 Oct 20;19:258. doi: 10.1186/s12974-022-02618-4 (PMC9583544; doi:10.1186/s12974-022-02618-4)
Supplement: Supplementary file 1 — Additional file 1: Tables S1 and S2. [file 12974_2022_2618_MOESM1_ESM.docx]

**Table SI**

| **Human Gene** | **Forward Primer Sequence 5' - 3'** | **Reverse Primer Sequence 5' - 3'** |
| --- | --- | --- |
| IL-1B | TACCTGTCCTGCGTGTTGAA | TCTTTGGGTAATTTTTGGGATCT |
| MCP-1/CCL2 | AGTCTCTGCCGCCCTTCT | GTGACTGGGGCATTGATTG |
| IL-6 | CAGGAGCCCAGCTATGAACT | GAAGGCAGCAGGCAACAC |
| TNF | CAGCCTCTTCTCCTTCCTGAT | GCCAGAGGGCTGATTAGAGA |
| UBA7 | TCTTTTCTCCAGGAAGAGAGC | GTAGGTGGCGGTGACAGTAG |
| UBE2L6 | GAGCATGCGAGTGGTGAAGGA | TCATGGGAGGCTTGAACGGA |
| HERC6 | GGAGCTGCCAGAACCAATTC | AGGCTTGTTGCTTTGCACTG |
| ISG15 | GGTGGACAAATGCGACGAAC | TCGAAGGTCAGCCAGAACAG |
| USP18 | TGGAAGTGAAGTCGTGCTGT | CAACCAGGCCATGAGGGTAG |
| HERC5 | TGGAAAATGACTGTGGACGCT | AGATCAAACGTGGGCCTCAAA |
| IRF3 | ACACATACTGGGCAGTGAGC | CCTACAATGAAGGGCCCCAG |
| IRF5 | CCTTACCTCTCCTGGGTTGG | TCCCCGTTGACCCATTGAAG |
| IRF7 | GCTACAAGCCCTCAGTCCAC | TGCCCTCTCAGGAGCCAA |
| IRF9 | AGAAAGGGGCGGAGAGATCA | CCACCCAGTTCCGGAGTTTT |
| GAPDH | ATGTTCGTCATGGGTGTGAA | TGTGGTCATGAGTCCTTCCA |
| **Mouse Gene** | **Forward Primer Sequence 5' - 3'** | **Reverse Primer Sequence 5' - 3'** |
| IL-1B | AGTTGACGGACCCCAAAAG | AGCTGGATGCTCTCATCAGG |
| MCP-1/CCL2 | CATCCACGTGTTGGCTCA | GATCATCTTGCTGGTGAATGAGT |
| IL-6 | GCTACCAAACTGGATATAATCAGGA | CCAGGTAGCTATGGTACTCCAGAA |
| TNF | CTGTAGCCCACGTCGTAGC | TTGAGATCCATGCCGTTG |
| UBA7 | GACGGGTCCTTGGAGATTGG | GCACGCTGTACTTCCTGAGT |
| UBE2L6 | AAAGCCTTCCAAGTGCGGAT | CAGGCTCTTCCAGATTCGGT |
| HERC6 | ATACGGCATAACGATCTGGACTGT | CCAGGTGACTTCGTCTGACTT |
| ISG15 | AGTCGACCCAGTCTCTGACTCT | CCCCAGCATCTTCACCTTTA |
| USP18 | TGAGATGTTTCGTCCAGCCC | GGTTGGCAGAACCTGACTGA |
| iNOS/Nos2 | GGAGCCTTTAGACCTCAACAGA | AAGGTGAGCTGAACGAGGAG |
| RANTES/CCL5 | TGCAGAGGACTCTGAGACAGC | GAGTGGTGTCCGAGCCATA |
| CD80 | TCGTCTTTCACAAGTGTCTTCAG | TTGCCAGTAGATTCGGTCTTC |
| CXCL10 | TTGAGATCATTGCCACGATGAA | TGGGTAAAGGGGAGTGATGGA |
| TWEAK | TACTGTCAGGTGCACTTTGATGA | TAAGATGAGCCCAGGGGAGG |
| Arginase I | GGCCTTTGTTGATGTCCCTA | ACAGACCGTGGGTTCTTCAC |
| CHOP | CCACCACACCTGAAAGCAGAA | AGGTGAAAGGCAGGGACTCA |
| Sxbp1 | CTGAGTCCGAATCAGGTGCAG | GTCCATGGGAAGATGTTCTGG |
| usXBP1 | CAGCACTCAGACTATGTGCA | GTCCATGGGAAGATGTTCTGG |
| GRP94 | AAGAATGAAGGAAAAACAGGACAAAA | CAAATGGAGAAGATTCCGCC |
| EDEM | CTACCTGCGAAGAGGCCG | GTTCATGAGCTGCCCACTGA |
| BiP | TTCAGCCAATTATCAGCAAACTCT | TTTTCTGATGTATCCTCTTCACCAGT |
| ATF4 | GGGTTCTGTCTTCCACTCCA | AAGCAGCAGAGTCAGGCTTTC |
| ATF6 | CACCCATCCGAGTTGTGAGG | GCAAACAACGTCGACTCCCA |
| VGLUT1 | TGCCCGTCTATGCCATCATC | GTGACGACTGCGCAAAAAGT |
| GAD1 | TTGGTTGCTACGGTGATGGG | CTCAGGATATGTCTGCTGGGA |
| VGAT | GGAGATAGCAGTCCTCGCTG | GACTTGTTGGACACGGAGGT |

**Table S2.**

| **Group** | **# Cingulate Cortex** | **# Thalamus** | **# Cerebellum** |
| --- | --- | --- | --- |
| Immunohistochemistry | | | |
| Multiple Sclerosis | 13 | 11 | 22 |
| Non-MS control | 6 | 7 | 7 |
| RNAscope | | | |
| Multiple Sclerosis | 4 | - | 5 |
| Non-MS control | 3 | - | 5 |
