# Supplementary figures and images for: ISGylation is induced in neurons by demyelination driving ISG15-dependent microglial activation
Source: J Neuroinflammation. 2022 Oct 20;19:258. doi: 10.1186/s12974-022-02618-4 (PMC9583544; doi:10.1186/s12974-022-02618-4)

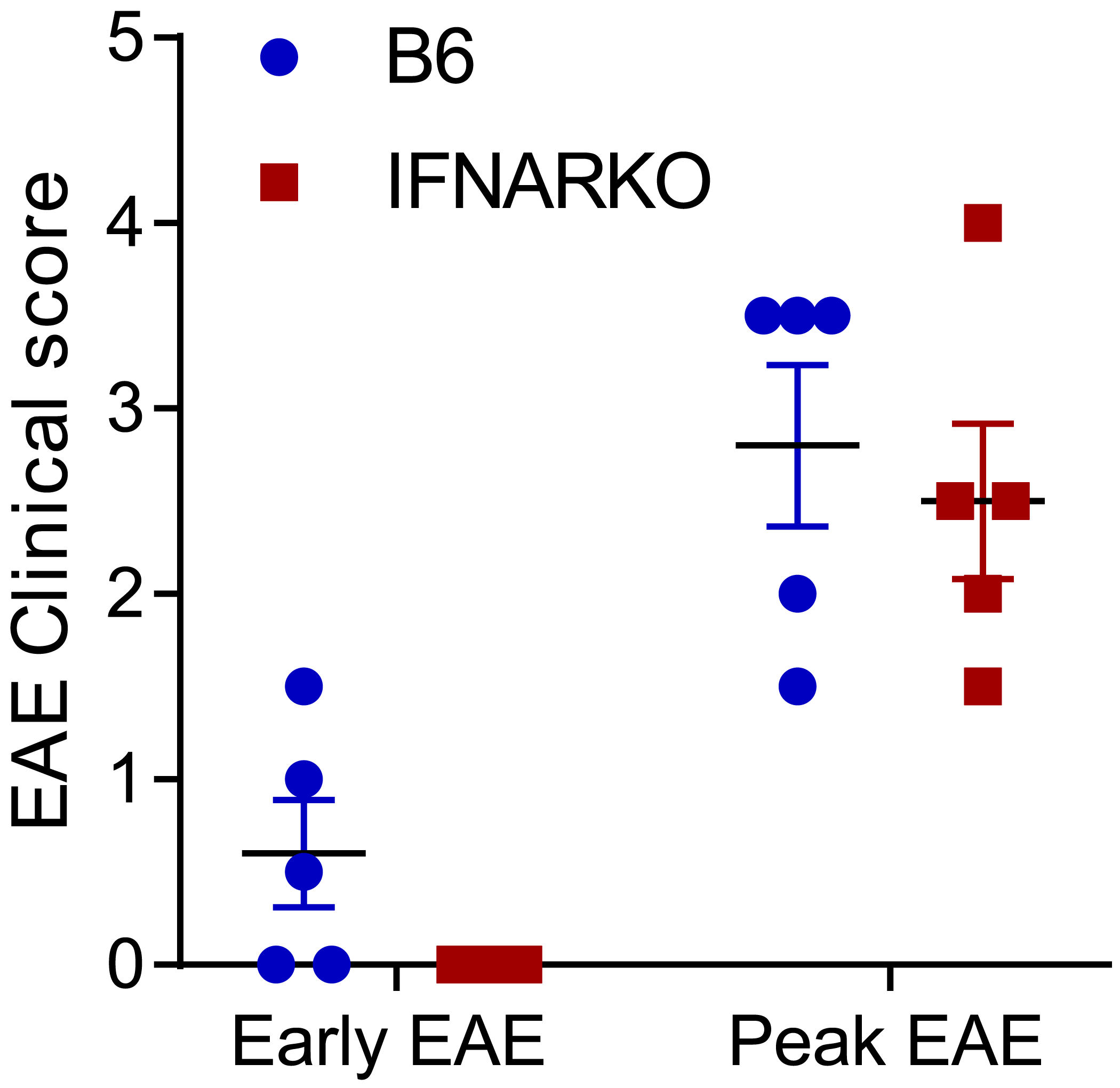

Supplement: Supplementary file 2 — Additional file 2: Figure S1. No difference in EAE severity between B6 and IFNAR KO mice. B6 and IFNα receptor knock out (IFNARKO) mice were immunized subcutaneously with 100 ug MOG35-55 and given 200 ng pertussis toxin on the day of immunization and 2 days later to induce experimental autoimmune encephalomyelitis (EAE). Clinical scores (detailed in methods section) were recorded daily beginning 7 days later. Mean clinical scores at 12 days post immunization (early EAE) and 18 days post immunization are plotted for both groups. Mean ± SEM are shown. [file 12974_2022_2618_MOESM2_ESM.tiff]

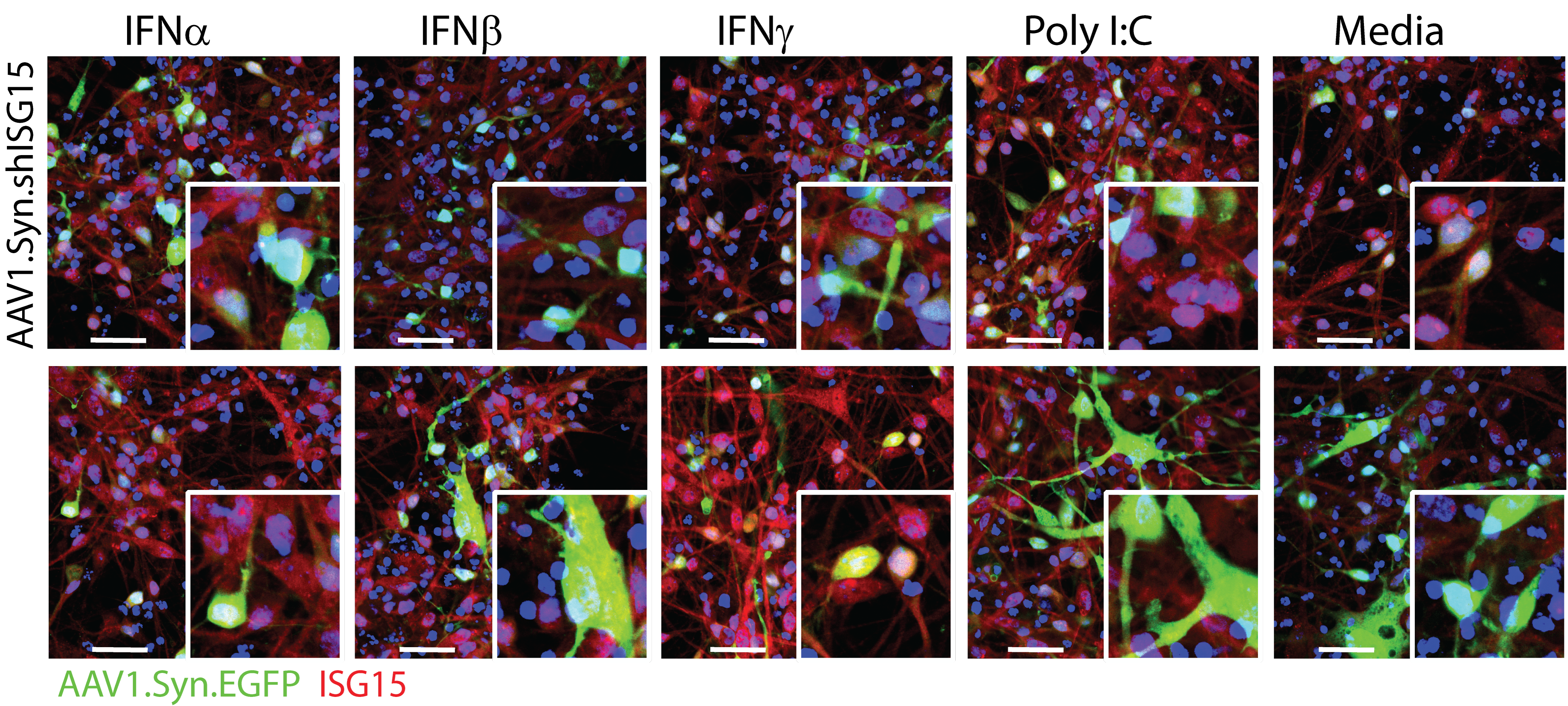

Supplement: Supplementary file 3 — Additional file 3: Figure S2. ISG15 induction in human neurons by IFN treatment. Human IPSC-derived neurons (E) were transfected with AAV1.Syn.EGFP with or without co-transfection with AAV1.Syn.shISG15 to silence neuronal ISG15 expression and 12 days later were treated for 24 h with 2000 U/mL IFNα, 100 ng/mL IFNγ or 2 ug/mL PolyI:C to drive ISG15 expression. Neurons were then fixed and immunostained for ISG15. Representative confocal images show ISG15 expression (red) in IFN-treated neurons (green) as well as non-neuronal cells. Neuronal ISG15 expression was silenced by AAV1.Syn.shISG15 co-transfection. [file 12974_2022_2618_MOESM3_ESM.tiff]

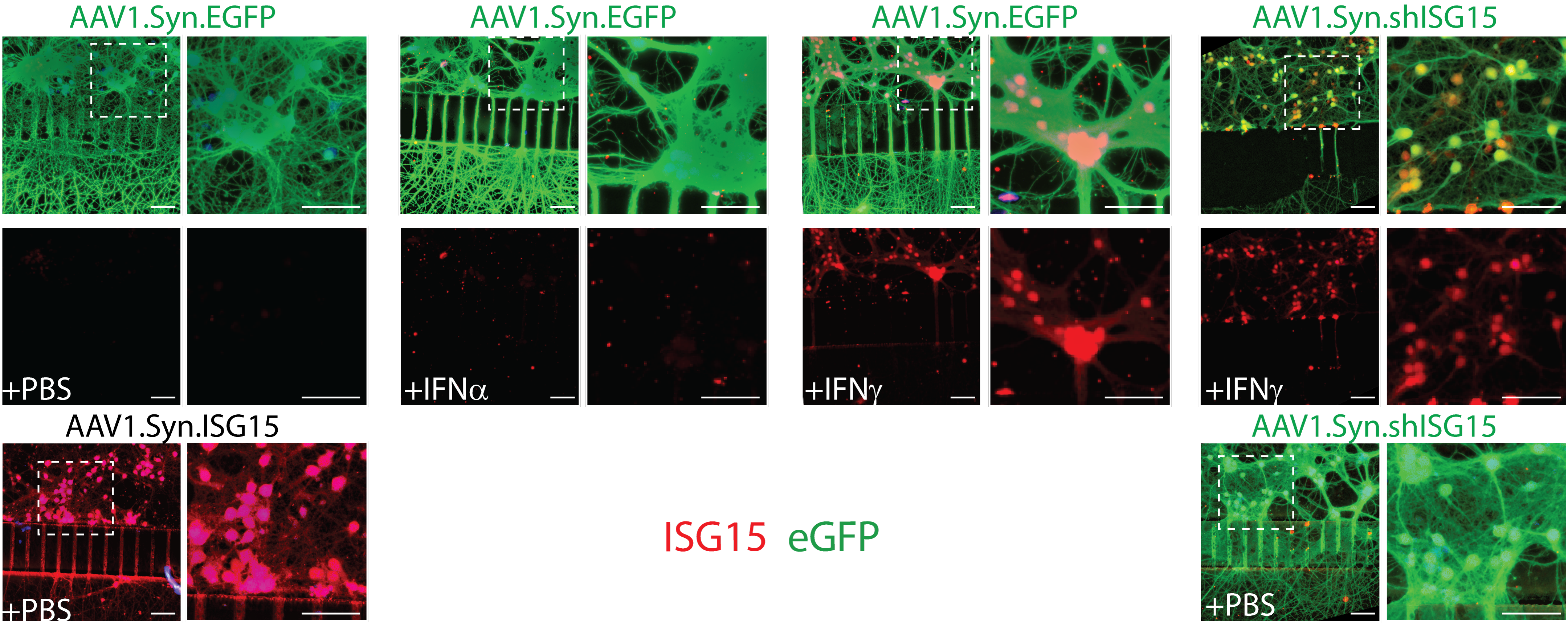

Supplement: Supplementary file 4 — Additional file 4: Figure S3. IFNγ but not IFNα causes retrograde induction of ISG15 in cortical neurons. Primary mouse cortical neurons were cultured in microfluidic axon isolation chambers and allowed to elaborate axons into the distal chamber. Neurons were transfected with AAV1.Syn.eGFP, AAV1.Syn.shISG15-eGFP as indicated. At DIV12 axon fields were treated with 2000 U/mL IFNα or 100 ng/mL IFNγ for 24–72 h and then cells were fixed and stained for ISG15 (red). DAPI-stained neuronal nuclei are shown in blue. Scale bars 100 microns. [file 12974_2022_2618_MOESM4_ESM.tiff]

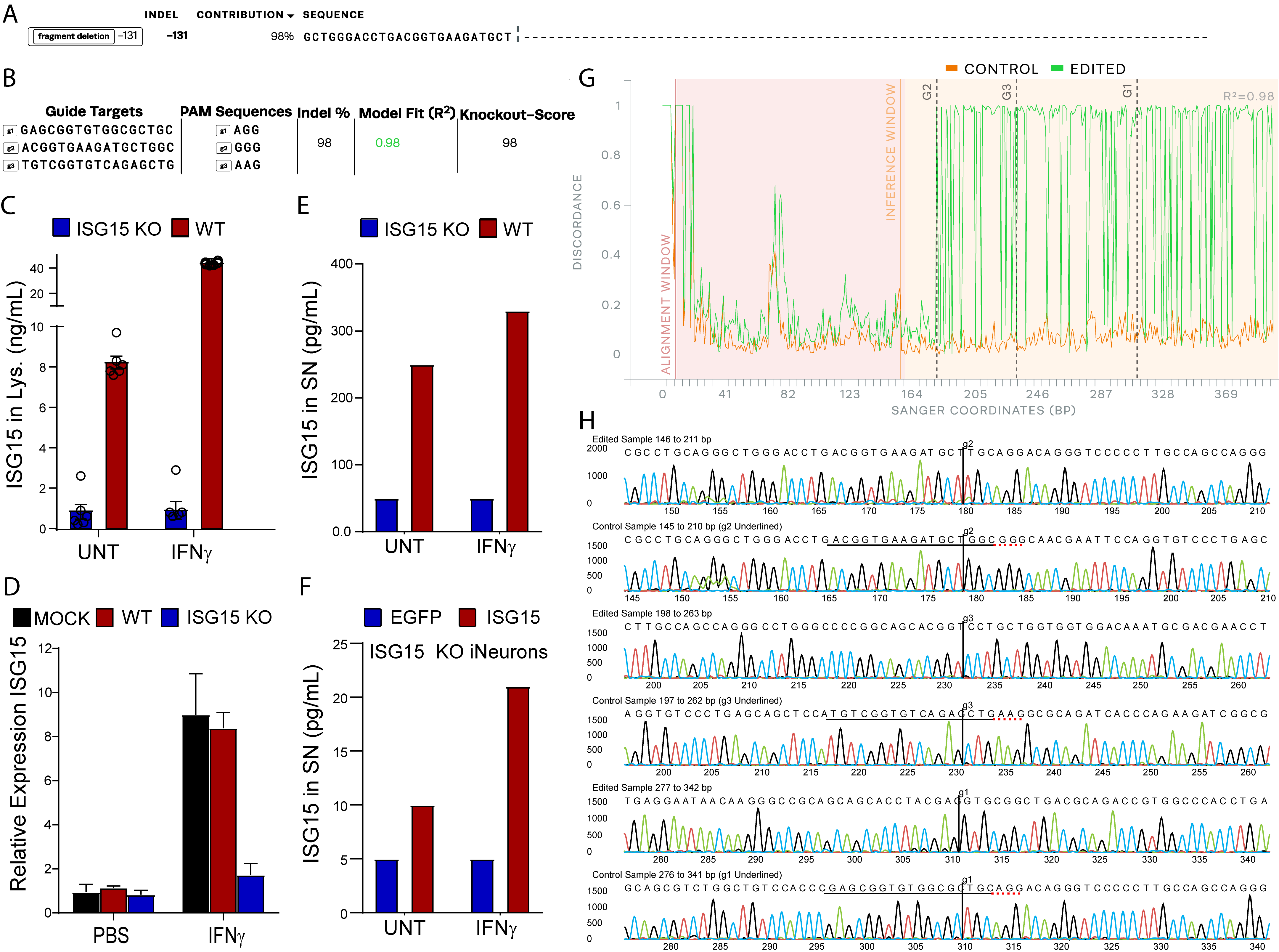

Supplement: Supplementary file 5 — Additional file 5: Figure S4. CRISPR/Cas9 gene editing of ISG15 in human IPSC-derived neural stem cells. Neural stem cells from human IPS cells were treated with three guide RNAs targeting ISG15 together with Cas9 complexes. Cells were diluted, plated, and allowed to grow out for 2 + weeks. DNA was then isolated and analyzed by Sanger sequencing. A) A single sequence exhibiting ISG15 gene truncation comprised > 98% of all analyzed sequences. B) Synthego ICE analysis showing guide RNAs and PAM sequences that were used for CRISPR-Cas9 editing along with % indel, model fit, and calculated knockout score. C-E) Following ISG15 knockdown, human IPSC-derived neurons or unedited parental cells were treated with 100 ng/mL IFNγ for 24 h as indicated and then we determined ISG15 protein levels in cell lysates (ELISA; C), ISG15 mRNA expression levels (RT-PCR; D), and ISG15 protein concentration present in cell supernatant (ELISA; E). F) We selectively restored ISG15 expression to neurons with AAV1.Syn.ISG15 transfection of ISG15 KO IPSC-derived human neurons and then treated these cells with 100 ng/mL IFNγ to measure neuronal ISG15 secretion. G) Discordance of “knockout” Sanger sequencing results with control ISG15 sequence showing expected ~ 25% concordance in target region. H) Sequence of ISG15 edited NSCs shown alongside the sequence from controls NSCs shows that discordance emerges at the guide 2 cut site (~ bp180). Mean ± SEM are shown. *P < 0.01 by unpaired Student’s t-test. [file 12974_2022_2618_MOESM5_ESM.tiff]

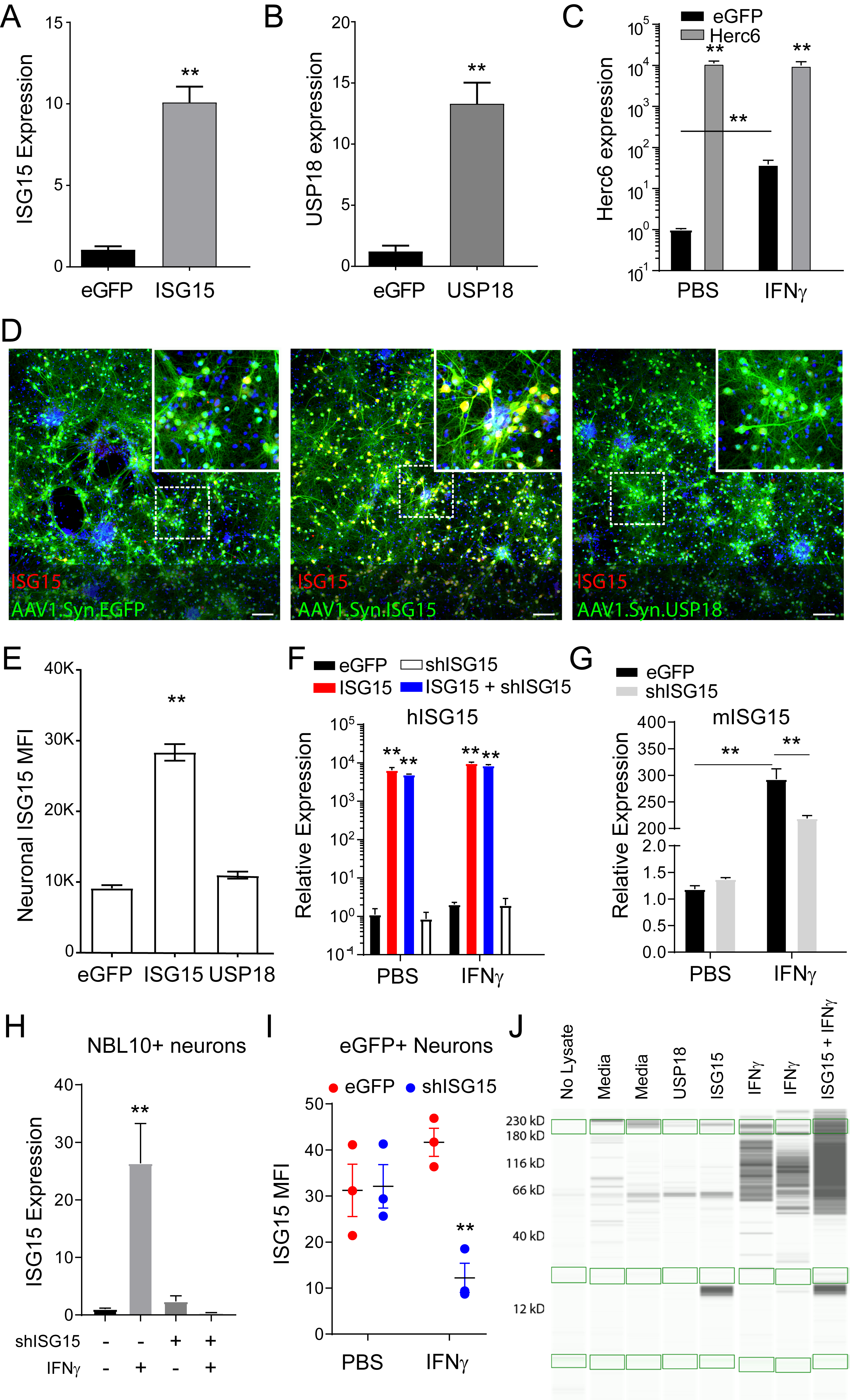

Supplement: Supplementary file 6 — Additional file 6: Figure S5. RT-PCR- and immunofluorescence-based validation of adeno-associated viral vectors for driving ISG15 and ISGylation regulatory proteins in neuronal cultures. To validate AAV-mediated expression of ISG15, and USP18, cortical neurons were infected at plating (DIV 0) with 2000 multiplicities of infection (MOI) of AAV1.Syn.eGFP control vector or each of the experimental vectors: AAV1.Syn.ISG15 (A), AAV1.Syn.USP18 (B), or AAV1.Syn.HERC6 (C) and mRNA expression determined by RT-PCR for the indicated conditions. D) The extent of neuronal ISGylation was elevated by AAV1.Syn.ISG15 as determined by immunofluorescent staining for ISG15 in each infection group (co-infected with AAV1.Syn.eGFP to identify neurons) quantified in ImageJ software (E). Murine cortical neurons were infected with AAV1.Syn.shISG15-eGFP or AAV1.Syn.eGFP control vector treated with IFNγ or PBS vehicle control. As shown IFNγ-treatment induced expression of endogenous ISG15 was incompletely suppressed by shISG15 (G), perhaps due to expression of ISG15 in non-neuronal cells such as astrocytes known to be present in these cultures. H) Neuron cultures infected with both AAV1.Syn.Cre-eGFP and AAV1.EF1alpha.NBL10 (which bear neuron-restricted HA-Tagged ribosomal subunits Rpl10) and co-infected with or without AAV.Syn.shISG15 were treated with IFNγ or PBS as indicated. We isolated neuronal ribosome-bound mRNA from these cultures using anti-HA.11 immunoprecipitation and performed RT-PCR to determine neuronal active translation of ISG15 transcripts. In these experiments, we found that neuronal induction of ISG15 translation was completely abrogated by shISG15. I) Cortical neurons were infected and treated as in G and then fixed, permeablized, and stained for ISG15. Images were acquired on an Axioscope. Mean fluorescence intensity of ISG15 stain in eGFP + neurons was determined using Image J macros. J) IPSC-derived human neurons were infected with AAV1.Syn.ISG15 or mock infected and trea [file 12974_2022_2618_MOESM6_ESM.tiff]

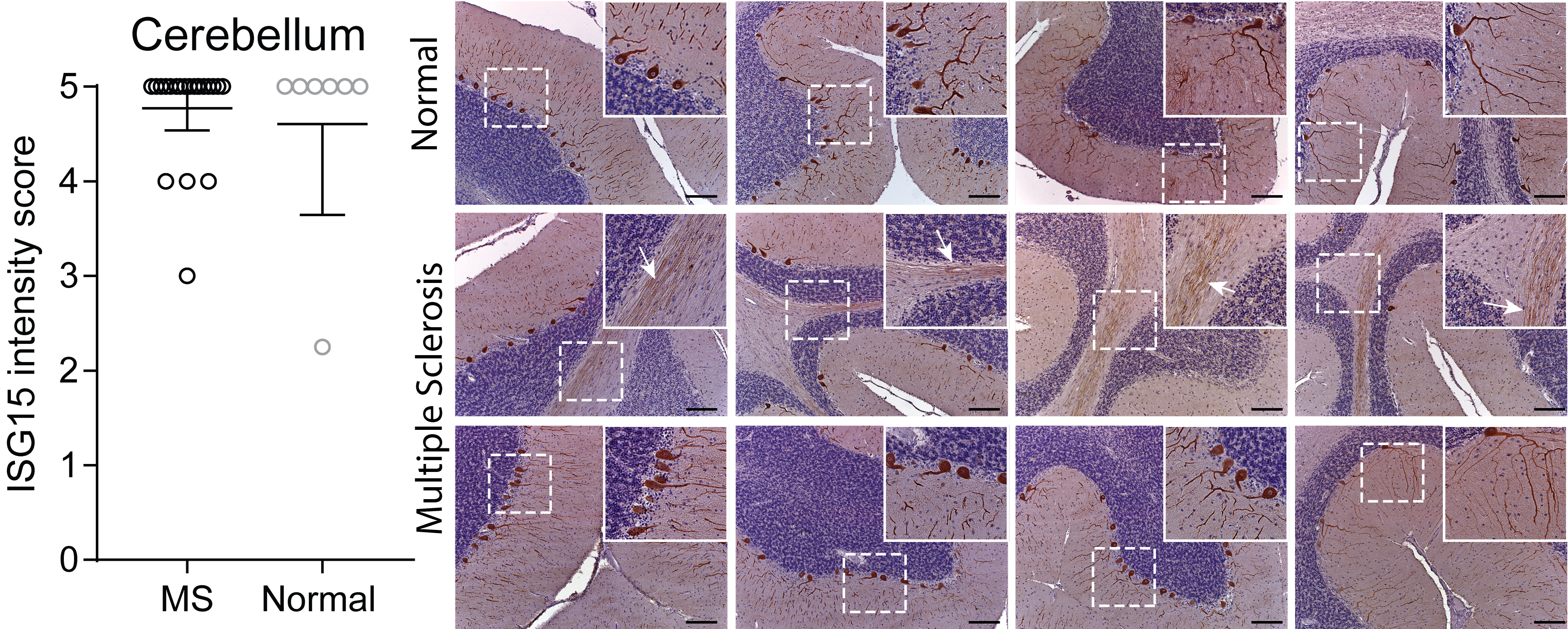

Supplement: Supplementary file 7 — Additional file 7: Figure S6. Axonal ISGylation in MS cerebellum. Paraffin-embedded MS postmortem brain tissue was obtained from Mayo Clinic tissue registry, Netherlands Brain bank, and Normal Aging Brain Collection Amsterdam (controls). Tissues were deparaffinized and antigen retrieved in 10 mM Tris 1 mM EDTA pH9.0 for 20 min at 95 C. Immunostaining is shown for ISG15 (brown). Tissues were counterstained with hematoxylin (blue). The extent of neuronal ISGylation was quantified in cerebellum (A). Representative micrographs are shown in B. Insets are digitally magnified. Arrows indicate areas of ISG15 staining on axons in cerebellar white matter tracts. ISG15 staining intensity scores are shown on left. Scale bar = 100 microns. Mean ± SEM are shown. [file 12974_2022_2618_MOESM7_ESM.tiff]

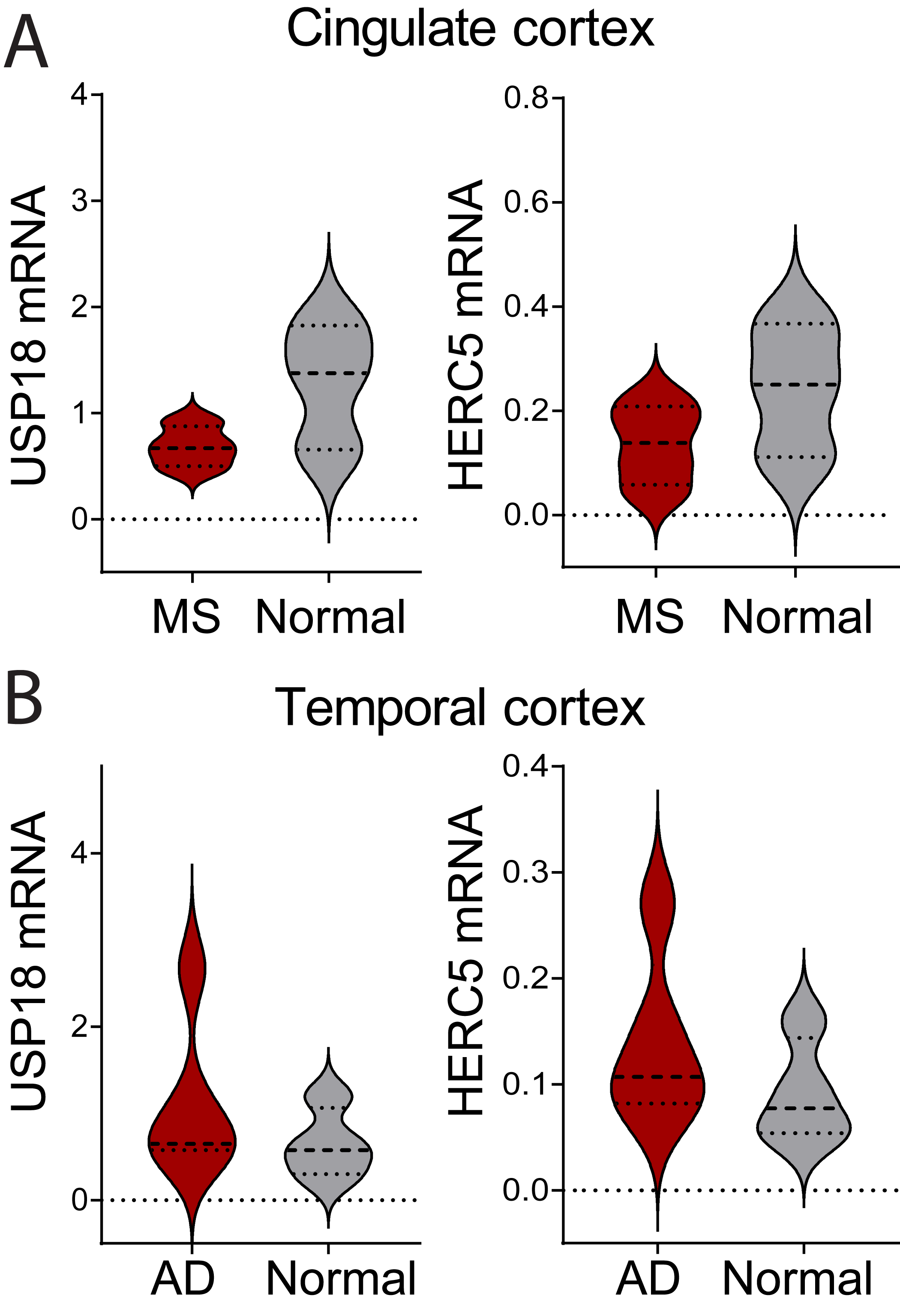

Supplement: Supplementary file 8 — Additional file 8: Figure S7. Top) Signal quantification of in situ hybridization with probes against USP18 and HERC5 in paraffin embedded temporal cortex tissue sections from patients with Alzheimer’s disease (AD; n = 5) and normal controls (NC; n = 4). Bottom) Similar quantification of in situ hybridization in paraffin embedded cingulate cortex tissue sections from patients with multiple sclerosis (MS; n = 4) and normal controls (NC; n = 3) [file 12974_2022_2618_MOESM8_ESM.tiff]
